# ASAS–NANP Symposium: Mathematical Modeling in Animal Nutrition: Opportunities and challenges of confined and extensive precision livestock production

**DOI:** 10.1093/jas/skac160

**Published:** 2022-05-03

**Authors:** Hector M Menendez, Jameson R Brennan, Charlotte Gaillard, Krista Ehlert, Jaelyn Quintana, Suresh Neethirajan, Aline Remus, Marc Jacobs, Izabelle A M A Teixeira, Benjamin L Turner, Luis O Tedeschi

**Affiliations:** Department of Animal Science, South Dakota State University, Rapid City, SD 57702, USA; Department of Animal Science, South Dakota State University, Rapid City, SD 57702, USA; Institut Agro, PEGASE, INRAE, 35590 Saint Gilles, France; Department of Natural Resource Management, South Dakota State University, Rapid City, SD, 57702, USA; Department of Animal Science, South Dakota State University, Rapid City, SD 57702, USA; Farmworx, Adaptation Physiology, Animal Sciences Group, Wageningen University, 6700 AH, The Netherlands; Sherbrooke Research and Development Centre, Sherbrooke, QC J1M 1Z3, Canada; FR Analytics B.V., 7642 AP Wierden, The Netherlands; Department of Animal, Veterinary, and Food Sciences, University of Idaho, Twin Falls, ID 83301, USA; Department of Agriculture, Agribusiness, and Environmental Science, King Ranch^®^ Institute for Ranch Management, Texas A&M University-Kingsville, Kingsville, TX 78363, USA; Department of Animal Science, Texas A&M University, College Station, TX 77843-2471, USA

**Keywords:** confined, digital monitoring, extensive, livestock production, modeling, sensors

## Abstract

Modern animal scientists, industry, and managers have never faced a more complex world. Precision livestock technologies have altered management in confined operations to meet production, environmental, and consumer goals. Applications of precision technologies have been limited in extensive systems such as rangelands due to lack of infrastructure, electrical power, communication, and durability. However, advancements in technology have helped to overcome many of these challenges. Investment in precision technologies is growing within the livestock sector, requiring the need to assess opportunities and challenges associated with implementation to enhance livestock production systems. In this review, precision livestock farming and digital livestock farming are explained in the context of a logical and iterative five-step process to successfully integrate precision livestock measurement and management tools, emphasizing the need for precision system models (**PSM**s). This five-step process acts as a guide to realize anticipated benefits from precision technologies and avoid unintended consequences. Consequently, the synthesis of precision livestock and modeling examples and key case studies help highlight past challenges and current opportunities within confined and extensive systems. Successfully developing PSM requires appropriate model(s) selection that aligns with desired management goals and precision technology capabilities. Therefore, it is imperative to consider the entire system to ensure that precision technology integration achieves desired goals while remaining economically and managerially sustainable. Achieving long-term success using precision technology requires the next generation of animal scientists to obtain additional skills to keep up with the rapid pace of technology innovation. Building workforce capacity and synergistic relationships between research, industry, and managers will be critical. As the process of precision technology adoption continues in more challenging and harsh, extensive systems, it is likely that confined operations will benefit from required advances in precision technology and PSMs, ultimately strengthening the benefits from precision technology to achieve short- and long-term goals.

## Teaser

Precision technology is rapidly being implemented in confined and extensive livestock production systems. Understanding what has been done is critical to successful short- and long-term gains in multifaceted and complex animal–plant–soil–water systems, requiring effective integration of precision system models.

## Introduction

### Global animal production settings

Climatic uncertainty, sustainable development goals, production efficiency demands, and the growing influence of consumer perception have presented significant opportunities and challenges for global animal livestock production in confined and extensive systems. These four factors provide motivation for change, while drastically increasing the complexity of animal production systems; producers simply cannot focus solely on traditional animal husbandry. The Intergovernmental Panel on Climate Change states that the “impacts of climate change on livestock productivity, particularly of mixed and extensive systems...are critical considering the very large areas concerned...” (IPCC, 2019). Livestock production is directly affected by climate change through increasing temperatures and precipitation variation, both of which influence water availability. This variation also influences animal production (weight gains and feed metabolism), reproduction (fertility), animal health (Rojas-Downing et al., 2017), and crop and forage production, and escalates heat stress (Baumgard and Rhodes, 2013; Roth, 2015; Neethirajan et al., 2018).

Increased greenhouse gas (**GHG**; e.g., CO_2_, N_2_O, and CH_4_) emissions are directly linked to inefficiencies in plant and animal production (Grossi et al., 2019). Efforts to slow and reverse climate change have led to the industry-specific GHG assessment including agriculture, putting livestock production under extreme scrutiny given it is the largest agriculture GHG contributor (FAO, 2021). Total GHG emissions from global livestock were 7.1 Gigatonnes (Gt) of CO_2_-equivalent/yr, representing 14.5% of all anthropogenic GHG emissions; cattle are the main livestock contributor (4.6 Gt CO_2_-equivalent/yr, 65% of sector emissions) with beef and dairy cattle generating similar GHG emissions (Gerber et al., 2013). In contrast, pigs, buffalo, poultry, and small ruminants have lower GHG emissions (each represents about 7% to 10% of the sector’s emissions; Gerber et al., 2013).

Despite GHG emissions from the livestock sector, the demand for meat and milk in 2050 is projected to increase by 73% and 58%, respectively, from their 2010 levels (FAO, 2011). As of 2018, there were 1.9 billion livestock units worldwide, specifically 965 million cattle, 242 million pigs, 237 million chickens, and 226 million sheep and goats (FAO, 2020). Since 1990, livestock units of cattle, buffalos, sheep, goats, and swine have increased by 16%, while chicken numbers have increased more than twofold (FAO, 2020). Regarding global meat distribution, Asia has been the largest producer since 1990, followed by Europe, North America, and South America, which collectively produces half the tons of meat compared with Asia. Given GHG concerns and increasing demand for meat and milk, 21st-century livestock production must address environmental concerns while simultaneously increasing production efficiency.

Livestock producers must also consider how to appeal to “conscious consumers” (50% of total consumers; Fromm, 2019) who value sustainably produced agricultural commodities while maintaining quality and taste characteristics. The demand for sustainably produced commodities presents an opportunity for the livestock industry to build consumer trust by demonstrating how livestock production may be a solution—not a driver—of climate change. Thus, enhancing active consumer outreach efforts to communicate environmentally sustainable production is imperative, especially when media presents information like “producing beef uses 20 times the land and emits 20 times the emissions” compared with producing beans (Aubrey, 2019), and “many environmental systems and processes are pushed beyond safe boundaries” by food production (Willet et al., 2019).

The livestock industry has already started to overcome some of these challenges. From 1977 to 2007, beef production modernization has resulted in reductions in total animals (30%), feedstuffs (19%), water (12%), land (33%), manure (18%), CH_4_ (18%), N_2_O (12%), and C (16%) while yielding a safer, more affordable product (Capper, 2011). Other research has helped appropriately contextualize the evaluation of sustainable production for major livestock types (dairy, swine, poultry, sheep/goat, and beef), highlighting the role ruminants have in utilizing marginal grasslands and restoring degraded cropland soils through livestock integration (Place and Mitloehner, 2012; Tedeschi et al., 2017a, 2017b; Kumar et al., 2019; Rotz et al., 2019; Menendez and Tedeschi, 2020). These complex and multifaceted problems—climate and the environment, production efficiencies, and consumer perception—require a systems approach to meet global animal production goals and to avoid negative unintended consequences; we cannot afford to trade one problem for another.

### Precision technologies and livestock production systems

Rapid advancements in precision technologies (software and hardware) have offered solutions to these complex livestock production challenges, mainly confined animal feeding operations. An important question is not if precision technology can facilitate solutions for these complex challenges, but rather how the tool of “precision technology” can be successfully implemented to achieve short- and long-term success for animal production. Confined animal feeding operations are production settings in which animals (e.g., swine and cattle) are in a centralized area. In these centralized areas animals are fed, watered, receive medical or growth treatments, reproductive procedures (e.g., artificial insemination), and are monitored for a specific amount of time or production phase (e.g., feedlot for beef cattle finishing). The centralization of confined operations means that resource inflow and outflow are centered around the animal production process. For example, whether grown on-site, in a surrounding area, or purchased and imported, feed is delivered to animals in confined operations. Similarly, waste products such as manure or wastewater are exported and treated or recycled. This makes confined animal operations high-throughput systems capable of supporting large animal populations and densities, which require intensive management to ensure maximum productivity and profitability. Because of this, confined operations generally provide living quarters and benefits for managers and employees; communication (cellular, Wi-Fi, satellite) support; adequate food, water, medicine, and shelter for animals; and accessibility to animals close to roads or other infrastructure. These features led to the early adoption of precision technologies in confined production settings, accelerating the generation of scientific knowledge and management experience in such systems that have only begun to be translated into extensive animal systems. In contrast to confined systems, extensive systems are animal production systems in which livestock do not depend on a centralized area for maintenance and growth, and often lack many of the infrastructure or resources available to confined operations. Extensive livestock productions systems often occur on rangelands, which occupy 54% (79.5 million km^2^) of the earth’s surface and are distributed globally (Rangelands Atlas, 2021), though many rangelands species composition has been altered from native to non-native or a combination of both. An estimated 91% of the world’s surface devoted to livestock production is composed of extensive rangeland systems (di Virgilio et al., 2018) with production concentrated in arid regions such as those found in Australia, the Middle East, Africa, South America, and North America (Robinson et al., 2011). Within the United States, forage-based livestock production is substantial with approximately 311 million ha of rangeland, 53 million ha of pastureland, and 16 million ha of hayland (USDA-NRCS, 2003; USDA-USFS, 2018) compared with 160 million ha of row-crop agriculture (USDA, 2019). However, not all of these forage production areas are extensive, ranging in size that typically expands as precipitation and, consequently, livestock capacity decreases. These extensive landscapes provide 70% of the feed demand for approximately 31.7 million beef cows, 3.8 million breeding sheep, and 2.2 million breeding goats in the United States (Sanderson et al., 2012; USDA, 2018a, 2018b) and offer a variety of ecosystem goods and services such as recreation, wildlife habitat, biodiversity, hydrologic function for ground water recharge, carbon sequestration, and open space for aesthetic value. Forage-based livestock production, including those on rangelands such as the beef cow/calf and sheep grazing systems, has not adopted precision agriculture technologies at the rates of other livestock sectors. Protein production on pastureland and rangelands is not conducive to intensive data collection because forage is harvested by grazing animals and not by machines on which sensors are easily mounted. In this production setting, animals must expend energy to gather nutrients in forage, water, mineral, and supplements often provided by management that are not necessarily centrally located across vast management units (e.g., >500 ha). Extensive systems typically have centralized animal handling facilities, but these are used infrequently for specific management needs (e.g., seasonal deworming or artificial insemination) due to associated costs and labor to locate and transport livestock, not for long-term holding. As such, precision technology adoption within extensive systems has increased challenges compared with confined systems, but, recently, advancements have made the deployment of these technologies more feasible for livestock research and production. At the center of these precision technologies are physical infrastructure (e.g., sensors) and cyber infrastructure (software, models, Internet of Things), capable of collecting high-resolution data that previously were too costly to measure. With increased capabilities from precision technologies for extensive systems, driving greater use, there exists a much larger need to ascertain which technologies to implement and how sustainable they are likely to be given the tremendous complexity inherent in extensive livestock production systems, which is only compounded with the integration of precision technology.

To handle this growing complexity, a “systems approach” is warranted (Turner et al., 2016; Tedeschi, 2019; Stephens, 2021). This approach accounts for the feedback mechanisms and time delays between interacting variables within a system, thereby improving the understanding of system drivers and identifying high-leverage solutions—where a small change in management has a significant impact (Sterman, 2000). To ensure that precision technologies are successfully integrated into livestock production, we must ask how models have been used and what type of models or combination of models are needed to benefit precision livestock production. 

Mathematical modeling (**MM**) is an essential component for precision livestock production. Jacobs et al. (2022, *companion paper*) describe the sustainable application of MM, which includes a comprehensive review of mechanistic, data-driven, real-time, empirical, statistical, artificial intelligence, deep learning, and machine learning models, highlighting their uses, advantages, disadvantages, and hybridization to handle biological processes and/or big data. Additionally, the authors emphasize the required modeling and programming skills for successful animal scientists both now and in the future. Modeling approaches are becoming increasingly integrated, so it is necessary to contextualize various types of models within precision livestock production systems to achieve model harmony. Model harmony suggests that MMs are developed, tested, and implemented in a way that not only maximizes the utility of precision technology to overcome challenges and meet goals but also contributes to and enhances the process of scientific critique, dialogue, and model refinement needed to accelerate the development of precision system models (**PSM**s) to inform precision technology adoption. In this current review paper, we focus on how models, particularly PSMs, fit into precision livestock systems, both confined and extensive, differentiating effective model types and their usefulness in the context of each system.

The practical requirements of making precision technologies sustainable for management are threefold. Firstly, precision technologies must achieve enhanced animal production. Secondly, its precision technologies implementation must lead to enhanced long-term economic viability (i.e., financial returns must be realized before the end of a product’s useful life). Lastly, precision technologies must create synergies from which producers can more easily connect “conscious consumers” to the production process and the environment (soil, water, plants, and wildlife). Therefore, the objectives of this paper are to 1) contextualize the role of models required for precision livestock farming (**PLF**) and digital livestock farming (**DLF**), 2) give specific examples of precision technology and PSM applications in confined and extensive livestock production systems, and 3) describe the future steps required to avoid paths that lead to failures to invest in long-term capacity for implementation of precision technologies (a behavior known as the “capability trap”) but instead lead to a synergy between research and development and livestock production industries.

## Precision Livestock Farming

As indicated above, the growth in the global population has led to increasing demand for animal products. Despite a loss in the total number of farms, the number of animals produced has increased. Many of these animals are produced indoors to decrease land use for livestock production and have greater control over environmental conditions. However, confined and crowded configurations make it difficult for farmers to closely monitor animal health and welfare (Helwatkar et al., 2014; Rowe et al., 2019), though levels and combinations of confinement practices may vary within and across operations. As climate change intensifies, the risk of disease, heat stress, and other health issues among livestock animals will increase (Bernabucci, 2019). This, in turn, will create a greater urgency to identify health issues and disease outbreaks as early as possible and take preventative measures to avoid large-scale economic losses (Thornton, 2010; Neethirajan, 2017). These issues, as well as escalating concerns over animal welfare, transparency, and environmental sustainability, have led to growing interest in digitalizing livestock agriculture through PLF technologies (Klerkx et al., 2019; Neethirajan, 2021a, 2021b, preprint, 2021c; Samperio et al., 2021).

The term ‘Precision Livestock Farming’ dates back to 1988, but it was not until 2000 that it gained significant traction when Daniel Berckmans reintroduced the term at a European Union conference, widely considered as the father of modern-day PLF. Berckmans (2017) stated that PLF consists of a “continuous automated real-time monitoring of production/reproduction, health, and welfare of livestock and environmental impact.” PLF technologies utilize process engineering principles to automate livestock agriculture (Benjamin and Yik, 2019). Examples of recent developments in PLF technologies include monitoring cattle behavior, detecting vocalizations such as screams in pigs, monitoring coughs in multiple species to identify respiratory illness, and identifying bovine pregnancy through changes in body temperature (Neethirajan, 2017). PLF technologies can also help farmers monitor infectious diseases within livestock agriculture, improving food safety and availability (Neethirajan et al., 2018). The use of PLF technologies will ultimately improve animal health and welfare while reducing food safety issues and maximizing efficient resource use (Norton et al., 2019). As the scientific literature on PLF and its industrial applications has evolved, both research and practice started to develop the concept of DLF (Neethirajan and Kemp, 2021a, 2021b) enabling complimentary or enhanced PSM applications. 

**Figure 1.**
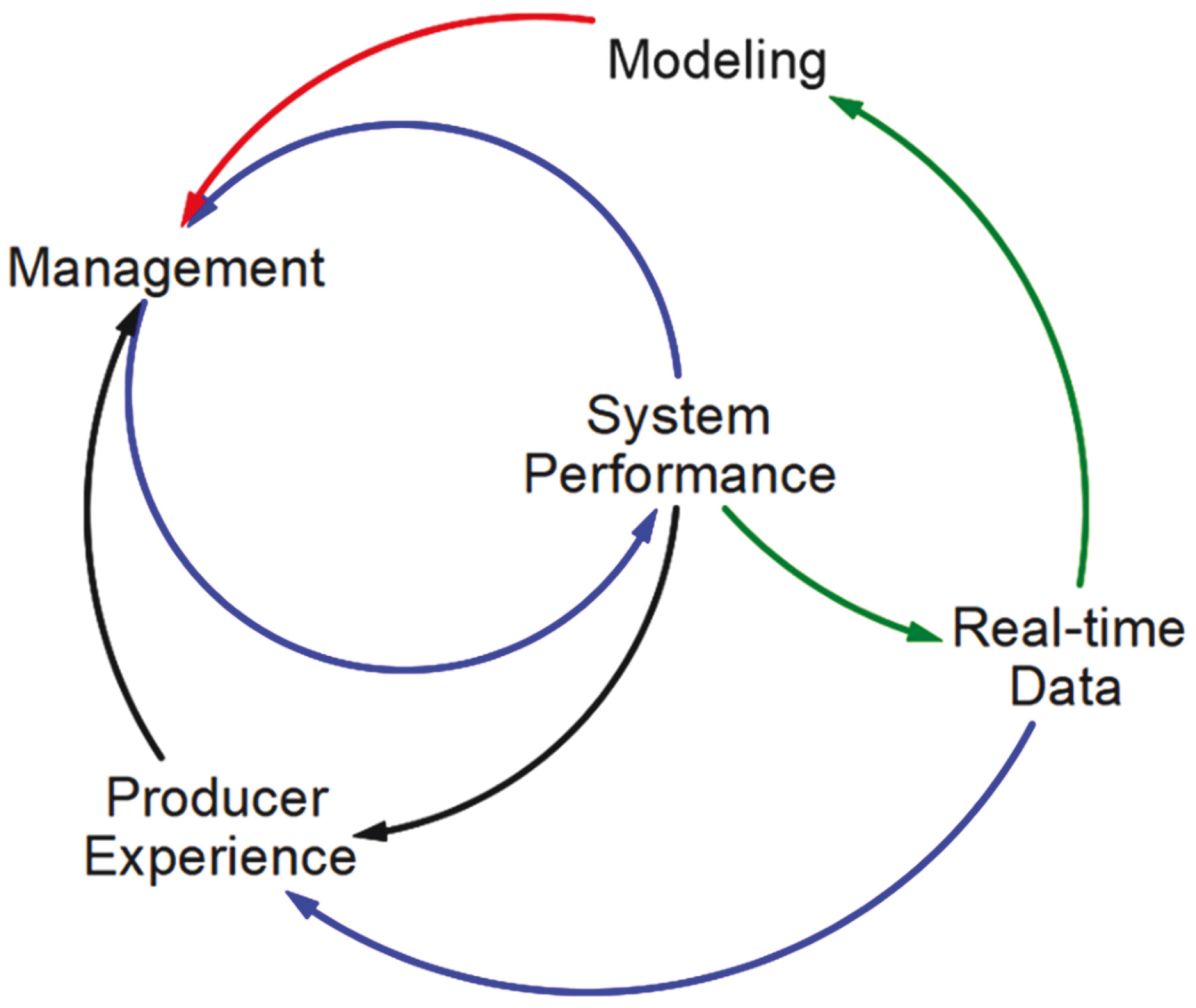
Diagram of the conventional producer decision process, including mental models (producer experience) and the role of modeling in relation to real-time data integration.

While PLF aims to maximize data collection to increase efficiency and productivity for farming and livestock management, DLF seeks to infer real-time data (i.e., “on the fly,” concurrently with reality) via predictive data modeling grounded in artificial intelligence and machine learning. Thus, DLF makes short-term automated management responses a near-term possibility and indicates a transition from reactive to predictive, and even prescriptive, capabilities. Instead of stepwise improvements by incrementally adding more data points, DLF introduces fundamental changes in the operations and value delivery that enhance the accuracy of processes and models in farm businesses. The removal of conventional platforms enables the necessary space for a digital acceleration powered by novel sensor devices, mechanisms, processes, and services to meet the demands of the new realities in modern animal farming. Hence, while PLF focuses on adding new technological functionalities, DLF also considers the context and complexities of the farming business (Figure 1).

### Precision measurement and management

Precision technologies can be separated into two major types: 1) measurement and 2) management. Precision *measurement* is any technology that increases a manager’s or researcher’s ability to increase the granularity of their data, for any metrics of interest, within an animal production system. Precision *management* includes technologies that replace or minimize the physical work and time that a person would typically require for specific livestock production tasks. Recently, precision technologies have expanded into virtual infrastructure that replaces physical infrastructure like fences. Although many papers highlight the potential impacts of these technologies (Berckmans, 2017; Morota et al., 2018; Fernandes et al., 2019; Tedeschi, 2019; Groher et al., 2020; Bailey et al., 2021; Tedeschi et al., 2021; Williams et al., 2021), their capabilities depend on the amount of data and level of granularity that is collected (e.g., millisecond vs. monthly), and requirements for communication (Wi-Fi, Bluetooth), power, human learning, maintenance, and infrastructure. Currently, most precision technologies are no more than simple alert systems; however, the industry is rapidly moving toward more complex applications.

### Implementation process for precision technologies

**Figure 2.**
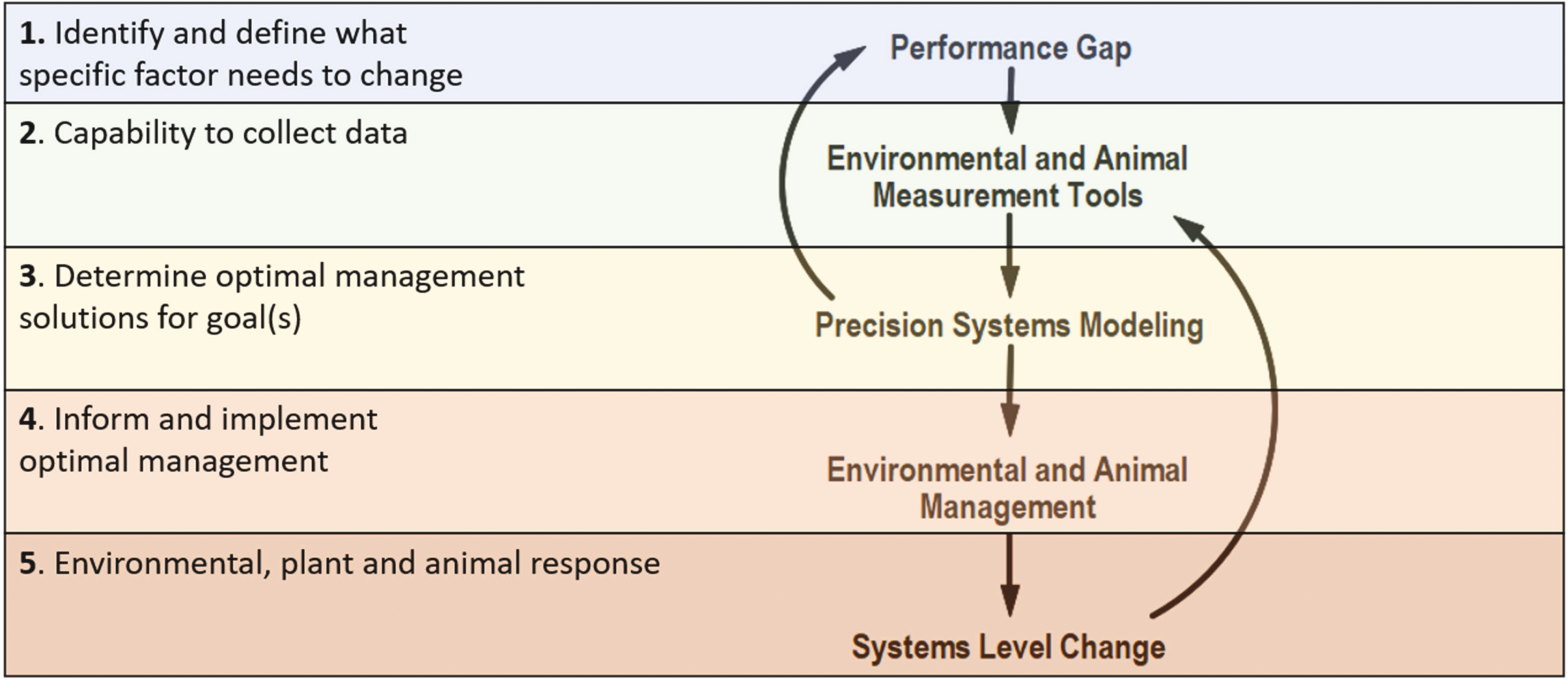
Conceptual diagram of five principles for sustainable precision livestock implementation using precision measurement and management tools integrated with mathematical models.

Understanding how to achieve sustainable implementation of precision livestock technologies requires a set of principles to filter through numerous potential technologies and applications and how they will integrate (or not) into existing systems. Application of the precision livestock principles (Figure 2) provides a logical and iterative process for sustainable and profitable implementation of precision technologies across confined and extensive systems. For this paper, we are defining the five principles for sustainable precision livestock implementation as 1) determining a performance gap, 2) increasing data collection and analysis capabilities, 3) determining the optimal solution with the aid of PSMs, 4) informing and implementing management changes, and 5) measuring systems-level responses and information feedback to remaining performance gaps (Figure 2).

#### Step 1: defining performance gaps

The first critical step requires management to identify the performance gap that needs to be closed if the goals are to be profitable and sustainable. The performance gap is defined as the difference between the current state of a livestock system’s performance and the desired state of performance (Gap = Desired – Actual; Sterman, 2000). Once the performance gap is understood, management must evaluate available precision livestock technologies and select the most appropriate precision measurement and management tool(s) required for altering production system processes aimed at closing the performance gap. This requires identifying which metric(s) or measurements will best inform management changes and, therefore, close the performance gap. Failure to evaluate trade-offs between available precision technologies and their strategic fit in a particular production system will likely result in collecting inadequate data, management frustration, and financial loss.

#### Step 2: increasing data collection and analysis capabilities

The use of precision measurement tools to collect data is the second step in the precision implementation process (Figure 2). Data collection implies that the appropriate technology has been identified to measure a specific metric at a given interval. Successful data collection requires a consistent timestep (e.g., daily) to ensure data quality. Humans, sensors, or robots may collect data. Each provides information about the status of a biological trait (e.g., body weight measured on a scale) or of its proxy (e.g., a surface measurement from an image as a proxy for body weight). Often measurements or proxies are combined (using modeling) to determine a metric that is not directly measurable by humans or precision devices (Jacobs et al., 2022, *companion paper*).

In considering precision measurement devices, it is important to consider the application and need for data storage and integration, data-processing algorithms, and potential sources of bias in data collection and algorithm development. Automated data collection provides a large volume of data. These data must be processed before being used and/or stored in a database in most cases. This includes cleaning, validating, and reconciling missing and abnormal values. If multiple data are being integrated simultaneously, which is often the case, the data must be further parsed and organized into a consistent format. This requires adequate governance, security, and meta-data, making data management one of the largest and most difficult challenges for precision data collection.

Given that many operations require the deployment of multiple technologies simultaneously, often manufactured by different companies or are custom made (Brennan et al., 2021; Tedeschi et al., 2021), effective data management and integration pose unique challenges to livestock production managers. In addition, researchers must consider the amount of data needed to capture the range of animal behaviors and generalize predictions to entire populations with sufficient frequency, quality, and precision. This challenge is increased in extensive systems where topography and resource heterogeneity may influence animal behavior differently across management units. Overall, selecting the most appropriate technique or technology for precision data collection and analysis is driven by the accuracy of estimation, the financial costs associated with the technology, and applicability and ease of integration into existing production settings.

#### Step 3: determining optimal solutions with aid of precision systems modeling

The third step of the precision implementation process involves the determination of optimal solutions with aid of PSM forms of MM. The purpose of MM is to make biological complexity manageable while overcoming the limitations and constraints of the human mind’s ability to process information (Sterman, 1994; Cronin et al., 2009; Turner et al., 2020). Precision livestock MM capable of handling large amounts of data (step 2) can be calibrated, tested, and optimized to identify feasible production changes needed to minimize the livestock system performance gaps (step 1). In agriculture, models are extensively used to capture pathways, test hypotheses, and identify high-leverage solutions, circumventing high risk, time constraints, or financial costs (Sterman, 2000; Turner et al., 2016; Tedeschi and Fox, 2020; Turner, 2020). Models have been applied to optimize diet formulation, livestock grazing dynamics, animal and plant performance/production, environmental impacts, and economic outcomes (Thornley and France, 2007; Turner et al., 2013; NASEM, 2016; Park et al., 2017; Tedeschi et al., 2019; Tinsley et al., 2019; Aderinto et al., 2020; Tedeschi and Fox, 2020; Taylor et al., 2022). Other examples include the Integrated Farm Systems model to conduct a life cycle assessment (Webb et al., 2020), the Ruminant Nutrition System that simulates nutrition and growth dynamics for different ruminant animal classes and production phases (e.g., steer feedlot finishing diet formulation) as well as economic-cost optimization based on feedstuff costs (Tedeschi and Fox, 2020), and the Ruminant Farm Systems whole-farm dairy system model that accounts for animal, manure, soil and crop, feed storage, and dairy environmental impacts (e.g., GHG; Kebreab et al., 2019; Hansen et al., 2021).

Models such as these are quickly moving from MM reliant on data exported from monitoring technology to real-time modeling capable of managing the smallest production unit(s) possible (Halachmi et al., 2019). Real-time data monitoring linked to dynamic-mechanistic MM models can execute tasks “on the spot,” processing data and yielding up-to-date predictions (Pomar et al., 2015, 2019; Pomar and Remus 2019a, 2019b). In addition, real-time models are more capable of technological integration relative to mechanistic MM grounded in historical data and, therefore, less likely to include the most up-to-date information. This has led to mechanistic models being considered difficult to apply because of the complex inputs and high-level knowledge required for proper model use (Ellis et al., 2019, 2020). Traditionally, MMs have been used to understand, illustrate, and support animal production (Ellis et al., 2020). However, their limitations have become more apparent over time as the velocity and volume of data have increased and the available data expand from numerical values alone to diverse sources such as image and audio files.

As the complexity represented in MM increases, so does their reliance on large databases (e.g., the National Academies of Science and Engineering and Medicine nutrient requirements and related nutrition equations; NASEM, 2016). Many national databases have application programming interfaces that enable them to be linked with MM, allowing for more rapid updates as the current body of animal nutrition knowledge advances (NASA, 2021; NRCS, 2021). Unfortunately, many published MM equations and parameters are difficult to reproduce (Jacobs et al., 2022*companion paper*), although they have laid the foundation of animal science modeling. This problem is exacerbated by older programming languages (e.g., Fortran) that are not intuitive and difficult to translate into updated models.

The potential dependence of databases should be considered when developing MM for precision livestock systems; in essence, it is a balance between data-hungry models and modeling capabilities. The challenge of automatically updating key input parameters highlights the growing role of real-time data collection (step 2). Applicability of MM is also influenced by the degree to which unmeasurable, yet significant parameters are reasonably accounted for, parameter variations in testing either meet or exceed observable ranges, and failure to consider non-measured parameters associated with real-time measures. Nevertheless, model choices about the type (mechanistic vs. empirical) and data resolution (historic vs. real-time) involve trade-offs between fidelity and efficiency.

It is important that the appropriate model or combination of models for PSM be used to minimize performance gaps (step 1). This requires that PSMs quickly obtain and integrate the various types of data from precision measurement devices and search for an optimal solution. This is one reason that most real-time models have a data-driven component (Table 1). It is also critical to understand why some livestock models can stay current and others become obsolete, such as over-reliance on large databases, so that integration with production systems does not overburden management with superfluous tasks outside normal model use and maintenance.

**Table 1.** Real-time models found in the literature using the search keywords: real-time, animal science, nutrition, and modeling

Author	Aim	Target	Type	Response
Hauschild et al. (2012, 2020); Remus et al. (2020c)	Provide daily tailored diets to individuals	Growing pigs	Gray box (empirical [data-driven] and mechanistic)	Diet composition to sustain observed growth
Peña Fernández et al. (2019)	Predict in real-time the indoor particle sizes concentration	Poultry	Data-based mechanistic	Predicted indoor particle sizes concentration
Parsons et al. (2007)	Integrated control of pig growth and pollutant emissions	Growing pigs	Data-based mechanistic	Predicted growth response based on diet intake
Stacey et al. (2004)	Control of broiler growth and nutrition	Broiler	Semi-mechanistic	Predicted growth response based on diet intake and control nutrient intake
Fu et al. (2020)	Predict diet energy digestion	Dairy cows	Kernel extreme learning machine	Predicted digestible energy and energy digestibility
Kashiha et al. (2013)	Report malfunctioning in a broiler house to the farmer in real time	Broiler	Empirical (data-driven)	Prediction of the distribution index of broilers
Gauthier et al. (2019); Gaillard et al. (2020b)	Provide daily tailored diets to individuals	Sows	Gray box (empirical [data-driven] and mechanistic)	Diet composition to sustain fetus development and milk production

#### Step 4: informing and implementing management changes

A MM capable of generating valuable information for making a management decision may be labeled a decision support tool (Figure 2). For a MM to be considered a decision support tool, it must pass a variety of mathematical, statistical, and logical tests in order that sufficient confidence can be placed in the MM to deem it suitable to inform management (Tedeschi, 2006; Turner, 2020). To pass such tests, MM performance is compared with the best available production data, descriptions of the production system, and managers’ experience and knowledge. The more confidence there is in the model to close the performance gap, the more likely the information is to be accepted and used.

Optimized solutions identified by the PSM (step 3 above) may be implemented with strictly management input, a mix of management input and automation, or be totally automated. Once implemented, the optimized solution(s) will alter the behavior and state of the livestock production system until the desired performance level has been achieved (step 1 above). However, changes in one element of an animal production system will inevitably lead to changes in other parts of the system due to their highly coupled, interconnected nature. Because of this, recognizing and monitoring both direct (intended) and indirect (unanticipated) outcomes throughout the production system are required for robust evaluation of the sustainability (or lack thereof) of chosen strategies.

#### Step 5: measuring systems-level responses and information feedback to remaining performance gaps

Successful implementation of precision measurement and management tools will likely result in changes to other parts of the system as a whole, such as changes in water quality, soil and plant health, carbon storage, biodiversity, and habitat conservation, among others (Figure 2). However, if optimized single-parameter management strategies are implemented and no system-wide feedback occurs (i.e., no synergistic feedback is generated across the production system), management should question if principles 1 to 4 (Figure 2 and described above) align with desired management changes. Although the adoption of precision technology makes livestock production systems more complex, it also facilitates synergistic activities aimed at diverse goals: enhancing animal productivity and production, regenerating environmental systems, and building consumer bridges. Effective implementation of precision technology systems, including successful integration with existing systems at the individual operation and industry level, requires an understanding of technology and time for management to learn and master its use before responses in environmental (sustainability) and financial (return on investment) metrics will be improved. In the following sections, we review examples of precision livestock production in confined and extensive operations to illustrate how the precision livestock implementation process described above pinpoints key challenges and opportunities in PSMs.

## Confined Precision Operations

### Case study 1: gestating sows

Within confined swine operations, the main challenge is to analyze the ever-increasing volume of data and use it in decision-making. The rapid evolution of techniques (e.g., machine-vision and feeders; Remus et al., 2020a, 2020b) suggests that these data could become available and affordable for pig farms soon (Piñeiro et al., 2019). For animals housed in large groups and with a short lifespan, constraints on the devices and data management are higher to obtain quality information. The “classical” information on growth and feed intake (and thus feed efficiency) are undoubtedly the most promising traits to be considered in pig nutrition because the feed cost comprises the largest part of the production cost. Information on body composition and traits related to health status are also important but need further development to scale up on commercial farms.

In the case of confined precision feeding, the decisions relative to nutrition or other management purposes are generally based on mathematical nutrition models designed to operate in real-time (Brossard et al., 2009; Hauschild et al., 2012; Andretta et al., 2016a, 2016b for growing pigs; Gauthier et al., 2019 for lactating sows; and Gaillard et al., 2019 for gestating sows). Artificial intelligence with the application of machine learning from historical data in combination with real-time data can also be used for the prediction of risk (e.g., risk of occurrence of health problem), events (e.g., ovulation), or performance (e.g., upcoming feed intake and milk production of a lactating sow) that can be used for the determination of nutrient supplies. Mechanistic models such as InraPorc (Dourmad et al., 2008) simulate the daily energy and nutrient partitioning in reproductive sows and were renewed for use in precision feeding for lactating (Gauthier et al., 2019) and gestating sows (Gaillard et al., 2019). These nutritional models calculate individual nutrient requirements and are dynamically connected to the flow of information provided by different sensors. This information then passes to the feeders that handle and implement the decisions to optimize nutrient supplies to each individual sow, each day.

Such an approach accounts for the large variability of nutrient requirements between sows in commercial farms, which stems from variability in performance, appetite, body condition, and changes occurring over time due to reproductive function (i.e., development of fetuses or production of milk, Gauthier et al., 2019; Gaillard et al., 2020a). For gestating sows, energy and nutrient requirements are calculated according to age, body weight (maintenance requirement), body condition at mating (requirement for body reserves), and expected litter size (requirement for conceptus) of the sow. The standardized ileal digestible lysine requirement is highly variable during gestation and varies with gestation stage, sow parity, and prolificacy (Gaillard et al., 2019).

The challenge in this approach is to get the information needed to calculate nutrient requirements. Body weight, physical activity of sows, and ambient temperature are required to calculate the maintenance requirement. Body weight can be measured at different times, for instance, when moving the sows from gestation to the farrowing pen. Backfat thickness, which is used in combination with body weight to determine the status of body reserves, can be measured simultaneously. The use of automatic weighing scales in the feeding stall allows much more frequent data to be obtained, and accelerometers can also be used to evaluate the physical activity of sows (Ringgenberg et al., 2010). However, this type of “mechanical” equipment may be challenging to maintain long-term, and video and image analyses may be a more promising, robust alternative for real-time evaluation of body weight (Cang et al., 2019), activity (Ahrendt et al., 2011; Labrecque et al., 2020), and perhaps body condition.

The interest in precision feeding strategies for gestating sows was evaluated through simulations by Gaillard et al. (2020b). In that study, a conventional 1-phase feeding strategy was compared with a precision feeding strategy, which consisted of mixing two diets with low or high nutrient content. The standardized ileal digestible lysine content was assumed to be 4.8, 3.0, and 6.0 g/kg feed and the protein content was 14%, 9%, and 16% in conventional feeding, low, and high diets, respectively. On average, the low diet represented 89% of the feed to be delivered by the precision feeding strategy. Compared with the conventional feeding, the average dietary standardized ileal digestible lysine content was 29.5% lower with precision feeding, while the average calculated dietary phosphorus content was 14.5% lower. The simulated proportions of sows that were given an excess or deficient supply of standardized ileal digestible lysine were reduced with precision feeding. Compared with conventional feeding, the precision feeding strategy allowed for a 3.6% reduction in feed cost per sow during gestation, and reduced nitrogen and phosphorus intake (by 11.0% and 13.8%, respectively) and excretion (by 16.7% and 15.4%, respectively).

### Case study 2: dairy cows

Dairy cows are selected for increased milk production, which has challenging aspects such as the high milk yield at drying off (Knight, 2005) and an increased feed price due to more concentrate feed (Kolver and Muller, 1998; García and Fulkerson, 2005). An increase in milk production requires more energy to be directed to the mammary gland, and this energy cannot be entirely provided via feed. Therefore, the cow needs to use her body reserves further and extend her negative energy balance period, negatively affecting pregnancy rates and health (Butler and Smith, 1989; Veerkamp et al., 2003; Pryce et al., 2004) and the farm economy. Hence, selecting high-yielding cows requires several management changes to solve these challenges.

Different strategies can be put in place to support milk production and reproductive performance. For example, increasing the frequency of milking from two to three times per day can increase milk production throughout lactation by about 10% to 20% (Pearson et al., 1979; Klei et al., 1997; Smith et al., 2002) and has been shown to increase milk production during the entire lactation (Erdman and Varner, 1995). However, it requires more working hours by the farmers or the use of automatic milking systems. In both cases, it reduces the time cows spend lying down or feeding, which are important activities for ensuring milk production and cow health (Gomez and Cook, 2010).

Another strategy is to delay the insemination day to a period during which the energy balance is back to positive (which is usually not the case for the typical 10 mo of lactation). This delay of rebreeding leads to an extended lactation that appears to be more advantageous, in terms of daily milk yield and economic profitability, for primiparous cows compared with multiparous cows (Arbel et al., 2001; Osterman and Bertilsson, 2003; Gaillard et al., 2016). This is probably partly due to the higher persistency of primiparous cows than multiparous, helping them maintain good milk production at the end of lactation. Regarding pregnancy rates, in most cases, no significant difference was found between cows in extended lactation and cows in a normal 10-mo lactation (Arbel et al., 2001; Gaillard et al., 2016).

Finally, the third option is to adjust the ration composition. Several feeding strategies have been experimentally evaluated and validated, like adjusting the energy content of the ration when the optimal proportion of concentrates is distributed (Jensen, 2014; Machado et al., 2014). Feeding a single ration to all the cows does not seem appropriate anymore as it could limit the expression of their milk potential (Bossen and Weisbjerg, 2009). Feeding cows according to lactation stage or even individually, for example, according to their energy balance, could potentially increase milk production. Thus, several individualized feeding strategies have recently been studied or are being evaluated, like the concentrate substitution rate strategy, defined as the reduction in feed dry matter consumption when the concentrate dry matter consumption increases. Maltz et al. (2013) worked on a weekly ration with a substitution rate of concentrates individually adjusted to the trough. Other experiments have been based on a single adjustment per cow at the end of the mobilization (Bossen et al., 2009; Alstrup et al., 2015). Milk production generally increases in the short term and sometimes throughout lactation (Bossen et al., 2009; Maltz et al., 2013). The effects of this individualized strategy also depend on the variable used for the adjustment (i.e., milk production vs. energy balance).

In this context, models can be useful to predict the consequences of different management strategies in terms of production and reproduction. The simulation of the distribution of nutrients through physiological functions and according to genotypes has been the subject of several models (Dumas et al., 2008; Friggens et al., 2013) with the aims to predict the performance of an animal and to help the breeders to make the best management decisions. This is the case of the GARUNS model, developed by Martin and Sauvant (2010), which considers the changing priorities of an animal during its life, and through repeated reproduction cycles. It has been tested and validated on cows of different breeds and parities (Phuong et al., 2015) and for different lactation durations (Gaillard et al., 2016). Looking forward, to developing, testing, and validating these models, data need to be collected through sensors or automatons (Cabrera and Fadul-Pacheco, 2021). Long-term, to obtain a PSM working in real time, data will need to be available almost instantaneously. Using wearable sensors and the Internet of Things, farmers will, for example, be aware of not only the productivity of each animal but also their health status. Therefore, they will be able to detect diseases such as mastitis or any other disease that can reduce milk production.

### Case study 3: mixed system—goats

Goats are one of the most adaptable livestock animals, and goat husbandry can be found worldwide in confined production systems or harsh, extensive environments. Both systems can benefit from the implementation of precision farming technologies, and in the last few years, some studies have been published regarding the applicability of PLF in goats (Puillet and Martin, 2017; Giger-Reverdin et al., 2020; Rao et al., 2020; Cellier et al., 2021; Su et al., 2022). Specifically, the implementation of precision livestock technologies in intensive/confined goat production systems is expected to happen earlier as the controlled environment makes it easier to meet the communication and infrastructure needs for installing and maintaining cameras, sensors, and other tools (Vaintrub et al., 2021). Such tools generate large datasets that need to be analyzed and interpreted to create benchmarks for phenotypic traits (Puillet and Martin, 2017). For instance, Abdelkrim et al. (2021) developed a perturbed lactation model in dairy goats incorporating representation of perturbations. This model is a valuable decision support tool as it allows the characterization of the potential milk production of a dairy goat (i.e., Saanen or Alpine) throughout lactation in a non-limiting environment as well as the depiction of the deviations induced by the on-farm conditions. The deviations represent the ability of the lactating goat to cope with environmental challenges. However, the main limitation of this model is the dependency on the data quality to avoid confusion between the deviation related to the environment and the low accuracy of the data recorded (Abdelkrim et al., 2021).

**Figure 3.**
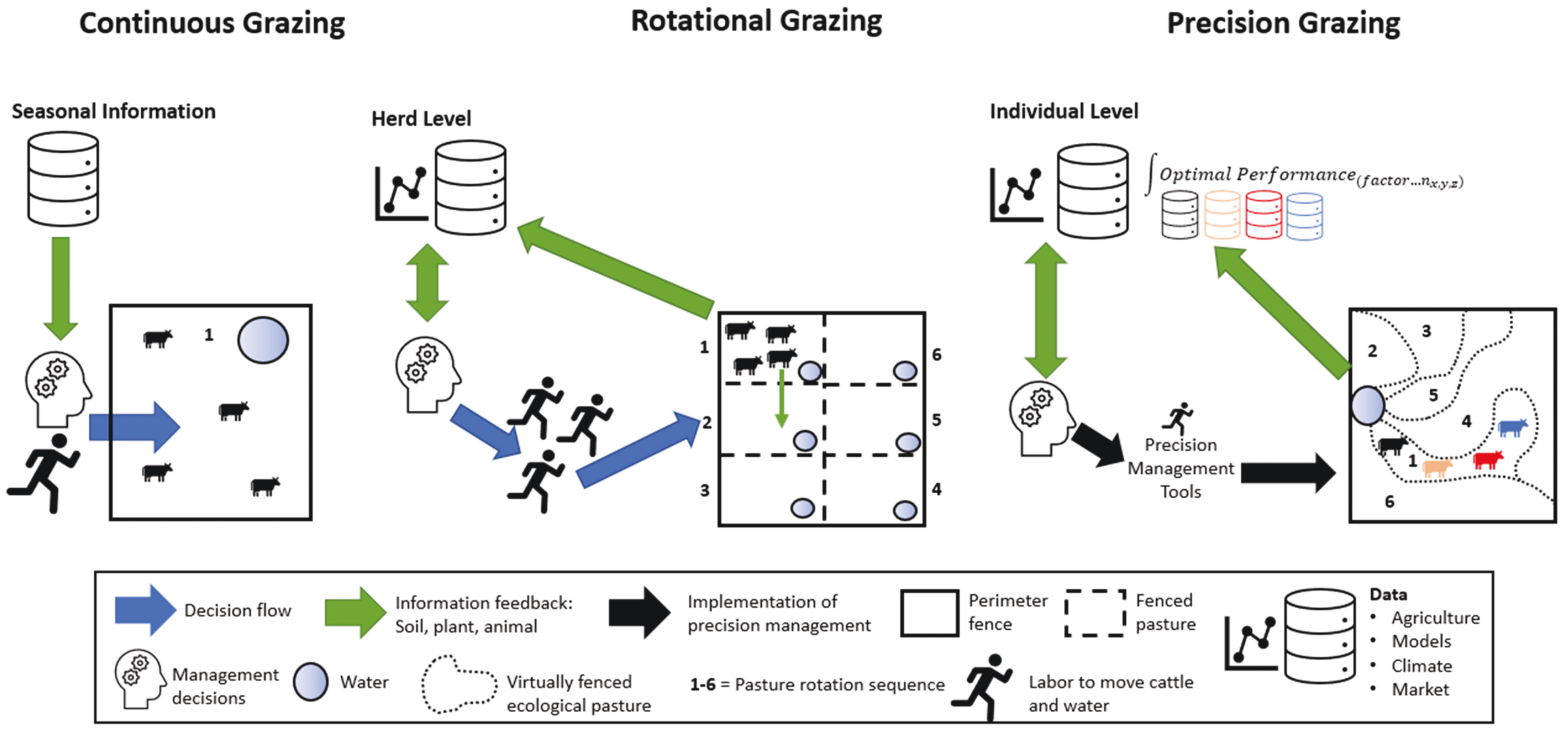
Continuous vs. rotational grazing as each relates to decision flow, information feedback, and management decisions, labor, and data.

In contrast, the benefits of implementing precision livestock technologies in extensive pasture-based goat production systems would not be immediate. However, these systems would benefit considerably from such technologies. The possibility of monitoring animals in remote locations without human interference would improve animal management related to predator attacks, health, and welfare issues, consequently resulting in significant labor reduction to monitor the herd (Aquilani et al., 2022). Efforts have been made in developing sensors with different suitability for grazing animals, for instance: using jaw activity to monitor feeding behavior (Navon et al., 2013), measuring heart rate and body temperature to determine predator attacks (Sendra et al., 2013), emitting auditory or electrical stimuli for virtual fencing (Marini et al., 2019; Aquilani et al., 2022), among others. Virtual fencing is one of the most interesting precision farming technologies for pasture-based goat farms as it replaces physical fences with virtual boundaries (Vaintrub et al., 2021). The use of virtual fencing would allow for the movement of animals according to forage availability and better pasture management that considers the soil–plant–animal interaction (Anderson et al., 2014; Figure 3). 

Despite a great potential for precision farming in goat production systems, this technology is still incipient, especially for extensive conditions. For example, the adoption of electronic sensors and measuring devices is substantially lower in dairy goat farms (48%) compared with dairy cattle farms (88%) in a study from Switzerland (Groher et al., 2020). Among the electronic sensors and measuring devices, the most adopted by dairy goat farms are the digital milk meter, milk temperature sensor, and electronic ear tags (Groher et al., 2020). In the same study, 70% of dairy goat farmers indicated that they do not adopt any electronic control devices (i.e., automatic feeding system, automatic kid feeder, and selection gates) and electronic data-processing options (i.e., a camera system for monitoring body condition score, pasture management, disease detection, estrus detection, data transfer into herd management systems, among others) compared with 28% of dairy cattle farmers (Groher et al., 2020). The low implementation of PLF in goat farms is likely due to the diversity of production systems, poor on-farm and/or on-field infrastructure for technological implementation—mainly in mountainous and remote areas, and high device costs—due to the need for miniaturization of sensors used for large ruminants combined with the high production costs of lower manufacturing amounts (Caja et al., 2020). Overall, the opportunities for increasing the adoption of PLF in goat farms rely on the development of interpretative tools to facilitate benchmarking (Puillet and Martin, 2017; e.g., PSM) and developing compact wearable devices, which still have a substantial life span and are wireless and robust enough to tolerate chewing by goats (common due to their inherent curiosity) (Caja et al., 2020).

## Extensive Precision Operations

### The importance of extensive livestock systems

**Figure 4.**
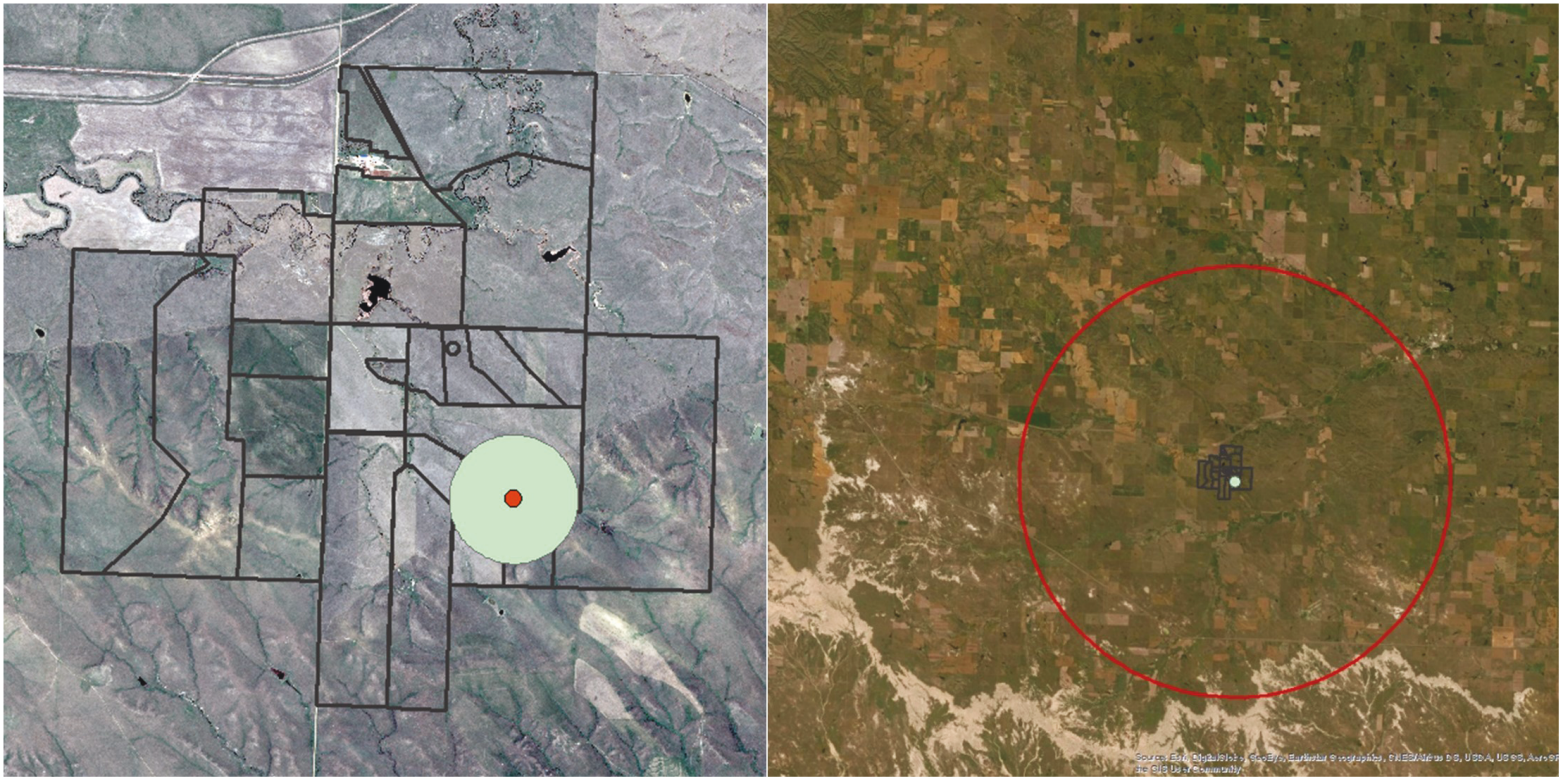
An example of the challenges of data transmission and range of data acquisition from base stations on extensive rangelands. The figure on the left is the potential range (400 m) of a Bluetooth 5.1 reader placed at a water source within a 72-ha pasture. The image on the right is the potential range (16 km) of a LoRa gateway over the same location. Other factors such as topography and line of site can affect data transmission range. Advances in cube satellite technology will enable data transmission globally between on-the-ground Internet of Things sensors and low earth orbit satellites.

The majority of advances in precision livestock management have been made in confined operations where greater control over animal nutrition and monitoring can be made. Many challenges exist in studying animals on rangelands due to the difficulty of accessing data across vast distances (~80 to 60,702 ha; Figure 4) with limited connectivity, heterogeneity of forage resources, and variable environmental conditions to which animals and technology are exposed. The most common grazing practice is continuous, or season-long, grazing in which livestock graze a single pasture for the entirety of the growing season without allowing the rest and recovery of the forage resource (Figure 3). Over time, animals tend to repeatedly graze preferred forage areas resulting in heavy grazing pressure, degradation of forage resources, and increased bare ground and undesirable plant species (Oates et al., 2011). In contrast, rotational grazing involves animals moving among divided paddocks to allow for the rest and recovery of forage resources following grazing events (Figure 3). Due to the higher stocking density within grazed paddocks, fewer preferred patches are grazed, which can increase desirable grasses, biomass production, and soil protection (Conant et al., 2003; Teague and Barnes, 2017). Precision technologies combined with real-time monitoring will likely result in whole system improvement in extensive livestock production systems such as rangelands.

### Case study 4: beef cattle

GPS technology has been used in beef cattle production systems to study animal movement and selection on extensive heterogeneous landscapes. This has given researchers and livestock managers valuable insight into the influence of forage quality, fire, topography, animal genetics, and management practices on livestock grazing distribution (Bailey et al., 2008, 2015; Zengeya et al., 2013; Augustine and Derner, 2014; Stephenson et al., 2017; Raynor et al., 2021). For example, GPS data from seven research stations across the United States demonstrated that topography alone could be used to predict grazing locations, with cattle utilizing lowland areas 120% more intensively than associated uplands. Additional factors such as steep topography and large distances to water can also greatly influence livestock grazing distribution, resulting in under- or over-utilization and degradation of specific sites (Bailey, 2005).

Traditionally, the application of this technology has been limited primarily to researchers within university and government organizations due to the high cost of commercially available GPS collars. Advances have been made to utilize “off-the-shelf” GPS tracking devices and open-source hardware solutions to reduce the cost to track animals (Knight et al., 2018; McGranahan et al., 2018; Karl and Sprinkle, 2019). In addition, GPS technology has been coupled with motion sensing technology such as 3-axis accelerometers that can help identify GPS locations associated with animal behaviors such as grazing, resting, and walking to better understand livestock behavior within extensive rangeland systems (Augustine and Derner, 2013; Brennan et al., 2021; Sprinkle et al., 2021a). For example, differences in grazing behavior can be used to identify differences in low residual feed intake vs. high residual feed intake cows, to ultimately select for animals that are better adapted to grazing rugged rangelands (Sprinkle et al., 2021b).

Despite GPS technology advancing knowledge of factors that drive livestock distribution on the landscape, applications in production settings have not been realized. For livestock, GPS and accelerometer monitoring devices often store data on board, requiring data to be downloaded and analyzed following deployment. A challenge with utilizing high-frequency accelerometer data is the large volume of generated data, making uploading information in real-time difficult. Edge computing accelerometers can reduce the data rate required by uploading less frequent machine learning model predictions based on raw sensor data inputs. Though the use of edge computing devices has been investigated to monitor animal behavior and health in sheep, no research has looked at deploying these devices on extensive rangelands over long periods (Kaler and Ruston, 2019; Vázquez-Diosdado et al., 2019). For these technologies to become a valuable tool for livestock producers operating on extensive grasslands, devices will need to incorporate communication technology and edge computing capabilities to reduce data transmission size and enable real-time tracking of animals.

As technological costs have come down for GPS technology, many commercially available options have become available for producers interested in tracking livestock. These devices can transmit livestock location using satellite communication technology or by sending data via long-range radio or “LoRa” communication to base stations (dos Reis et al., 2021). The availability of real-time GPS tracking opens many applications to livestock producers. The simplest example may include algorithms to detect and alert producers when animals are outside of pasture boundaries or remotely locate animals in areas where rough terrain or travel distance limits frequent opportunities for livestock managers to observe cattle welfare visually. More advanced algorithms have been used with GPS and accelerometer sensors to accurately predict livestock behavior and calculate metrics such as individual daily time spent grazing (Brennan et al., 2021). Variability in movements and behavior associated with GPS-tracked livestock can be an effective way to monitor livestock welfare concerns such as water failure, disease detection, or changes in behavior linked to distress or parturition (Tedeschi and Menendez, 2020; Tobin et al., 2020, 2021; Fogarty et al., 2021).

GPS and accelerometer-based sensors are an example of precision measurement technologies that can be used to better understand animal movement and behavior. With the advent of precision management technologies such as virtual fencing, measurement technologies that can identify and move animals within extensive landscapes to improve natural resource management and nutrient capture can be incorporated into PSMs. For example, metrics of association patterns and distance traveled among cattle within a herd may indicate animals searching for more palatable forage (Tobin et al., 2021), which in turn can be used to virtually rotate animals to a new paddock.

Among one of the most promising applications of precision livestock management within extensive systems is the integration of remotely sensed satellite imagery data to inform virtual fencing rotations. The democratization of satellite imagery has generated a wealth of free or low-cost imagery, enabling new ways to study, observe, and measure the earth’s natural systems. This is especially true in extensive grasslands where remote sensing greatly improves our ability to study and understand complex ecological interactions across the landscape, allowing assessment at landscape-level scales compared with traditional point-based assessments (Ramoelo et al., 2015; Yu et al., 2018). As technology advances, monitoring of rangeland vegetation via remote sensing platforms will facilitate research products freely available to land managers (Browning et al., 2015). The value of remote sensing to measure and monitor grasslands has been well documented including modeling seasonal changes between above-ground biomass and individual pasture phenology (Wang et al., 2019), estimating biomass across different pastures and plant communities (Primi et al., 2016; Otgonbayar et al., 2019), and estimating paddock grazing capacity (Phillips et al., 2009).

Precision measurement data derived from satellite or drone imagery can also be used to monitor forage composition, quantity, quality, and grazing intensity on the landscape through time (Goodin and Henebry, 1997; Todd et al., 1998; Ausseil et al., 2011; Franke et al., 2012; Zengeya et al., 2013; Ramoelo et al., 2015; Figure 3). The ability to map forage metrics based on remotely sensed imagery could be a powerful tool for making data-driven decisions about paddock rotation to maximize forage nutrient capture for grazing animals and minimize the deleterious effects of overgrazing on the system (Jones et al., 2021). Other precision technologies like real-time weighing may be used synergistically with remotely sensed data to more precisely adjust stocking rates relative to forage resources and animal requirements. Real-time weighing is possible using precision walk-over scales located within pastures that measure daily individual weight. These weight data have the potential to improve model inputs when using equations like net energy for gain (NASEM, 2016; Figure 3) instead of an estimated body weight. Thus, incorporating virtual fencing technology into precision pasture management allows land managers the ability to draw fence boundaries based on environmental factors that are derived from other precision measurement technologies (e.g., remote sensing) such as elevation, soil type, plant communities, animal weight, and forage quality, giving greater control over animal movement on the landscape and potentially allowing animals to capture the highest quality forage (Figure 3).

### Case study 5: sheep

Grazing sheep in extensive systems offers the ability to produce food and fiber with low input costs. Precision technology has a role in sheep management in extensive systems to enhance food and fiber production. Nutrient requirements change based on the production stage, the number of fetuses a pregnant ewe is carrying, and the desired growth rate of lambs (NRC, 2007). Utilizing technology, remote drafting systems can be used to provide precise supplementation to individual animals without the requirement of sorting sheep into groups and multiple housing locations for each group (Jordan et al., 2006). Additionally, precision feeding had an estimated gross margin of AU$6,000 and improved the reproductive success of a flock (Jordan et al., 2006). Bowen et al. (2009) evaluated the accuracy of a remote, solar-powered drafting system to supplement grazing Merino wethers. Treatment groups were allowed access to ad libitum lupin grain 1, 2, 4, or 7 d a week through an automated drafting gate at the water source. Based on their radio frequency identification tag and assigned treatment group, the remote drafter allowed access to supplement or sorted the sheep back to pasture. Sheep were accurately given access to the supplement yard through the automated gate with only 2.1% of incorrect drafts into the self-feeder (Bowen et al., 2009; Brown-Brandl et al., 2019).

Virtual fencing systems have been investigated to control the movement of grazing sheep (Jouven et al., 2012; Marini et al., 2018). Marini et al. (2018) assessed the use of commercial dog training units (Garmin Ltd., Olathe, KS, USA) to keep Merino wethers out of an exclusion zone within a paddock. The exclusion zone was comprised of sandy soils with a greater likelihood of erosion. Virtual fencing effectively controlled a small group of sheep (*n* = 6) within a 20 × 80 m paddock. Additionally, by day 3, sheep were deterred from the exclusion zone by an auditory warning cue prior to electrical stimulation (Marini et al., 2018). Unfortunately, results vary on the efficacy of virtual fencing systems for sheep. Jouven et al. (2012) also found that virtual fencing was effective on small groups of sheep (*n* = 5), but when applied to larger flocks (*n* = 35), it could not replace traditional fences for complete control. The challenge with sheep is their naturally gregarious nature. Marini et al. (2020) found that at least 66% of the flock needed virtual fence collars for effective control but suggested further studies are needed based on the small sample size (*n* = 9).

Modeling can assist in optimizing the management strategies of invasive species that sheep and goats can manage. Chalak and Pannell (2012) utilized stochastic dynamic simulation modeling to understand the most efficient and cost-effective solutions to managing weed blackberry (*Rubus anglocandicans*), an invasive shrub found worldwide. These modeling techniques were used to determine the estimated net present value of various control measures across different infestation levels. Results showed that control strategies were highly dependent on labor cost and method, and infestation level. Similar models could be applied to other plants commonly managed by small ruminants and potentially enhanced using precision management technologies like virtual fencing. However, if collars are required for nearly every animal, the cost of the system for a commercial operation may be a concern, especially given the standard five sheep per one cow–calf pair animal unit equivalent on grazing land.

Precision technology combined with machine learning can also quantify changes in sheep behavior to assist in monitoring health and well-being (Mansbridge et al., 2018; Vázquez-Diosdado et al., 2019). Using accelerometer and gyroscope sensors on ear tags and collars of sheep (*n* = 6) paired with various machine learning algorithms, Mansbridge et al. (2018) were able to classify the eating behaviors of sheep. Both ear and collar locations could distinguish between grazing, ruminating, and non-grazing behavior, saving time and reducing cost and human error of traditional observation. Random forest models had the greatest accuracy for behavior classification for both ear (91%) and collar locations (92%). Confusion matrices indicated that the prediction of all three types of behavior had overall performance values of 86% and greater. The combination of accelerometer and gyroscope features was likely a contributing factor to increased accuracy given both technologies’ greater number of features.

Accelerometer and gyroscope sensors were also used on the ears of differing ages, breeds, and body conditioned sheep (*n* = 26; Vázquez-Diosdado et al., 2019). Walking, lying, and standing behaviors were classified with online and offline algorithms. Additionally, research technicians visually timestamped behaviors with computer synchronized stopwatches for 2 h in the morning and 1 h in the evening. Results indicated that a combined algorithm of offline *k*-nearest neighbors and online *k*-means was most accurate in predicting behavior. The greatest precision was for predicting walking (92.9%), while the least accurate was for predicting standing (78.4%), with an overall accuracy of 85.2%. Discrepancies in precision technology and actual observed behavior exemplify the need for continued research and validation of technology and modeling for sheep production. However, this study showed that using both online learning algorithms and offline trained classification approaches can accurately identify sheep behavior (Vázquez-Diosdado et al., 2019).

## Discussion

Precision technology has the potential to result in significant improvements in efficiency in livestock production systems, both confined and extensive, and accelerate animal science research across a range of subdisciplines ranging from animal health, nutrition, reproduction, and behavior. The five principles outlined in the precision livestock implementation process will aid both technology adopters and scientists to ensure their efforts to collect and utilize appropriate data and PSM will achieve desired animal performance improvements. Tremendous value exists in using the five principles for sustainable precision livestock implementation to better understand and utilize “big data” through precision technology integration. In confined systems, precision feeding is commonly used to close the performance gap in feed use and harness individual animal potential. Likewise, in extensive systems, GPS and virtual fencing provide opportunities to close the gap between animal performance gains and resource utilization.

Precision tools required to capture key metrics must do so non-invasively to individual animals at costs low enough to facilitate economies of scale. Within extensive systems, precision tools need to be autonomous and not require significant maintenance and human intervention, given that most precision technology is secured to an individual grazing animal that may be grazing for months at a time between gatherings. Therefore, the effective use and implementation of PSM in both confined and extensive systems require aligning technology implementation and data storage and processing with management expectations for cost, payback period, and time and effort required to maintain the technology. Identifying and communicating challenges in automated implementation are required to enhance effectiveness. For instance, precision applications between species (cattle, swine, and goats) must be customized to increase the practicality of use. Adopting a systems-level approach to precision livestock management will support the successful identification of performance gaps, implementation of measurement and management tools, and PSM development.

Realizing optimal PSM solutions that are feasible and practical in confined systems will result from increasing function and reliability, such as balancing individual rations, milk production, and lactation duration. In confined systems, improvements in precision feeding will likely decrease input feed requirements and increase nutrient cycling from manure. In extensive systems, PSM can balance optimal grazing rotations with energetic efficiency from differences in distance traveled for forage and water (including use of strategic or precision supplementation), so long as data communication challenges to inform PSM are overcome given the extant geographic nature of extensive systems, highlighting the importance of selecting the correct variables and avoiding unnecessary data collection. Extensive operations aim to increase resilience to climatic variations and maintain forage production, quality, and water quality, thereby decreasing nutrient variation and economic risks of nutrient shortages (e.g., selling due to drought). In either case, successful applications will increase confidence in precision solutions to inform and implement autonomous livestock management in both confined and extensive systems, where the health and welfare of livestock stand to be improved as individual animal data are collected and models provide additional insights over time.

**Figure 5.**
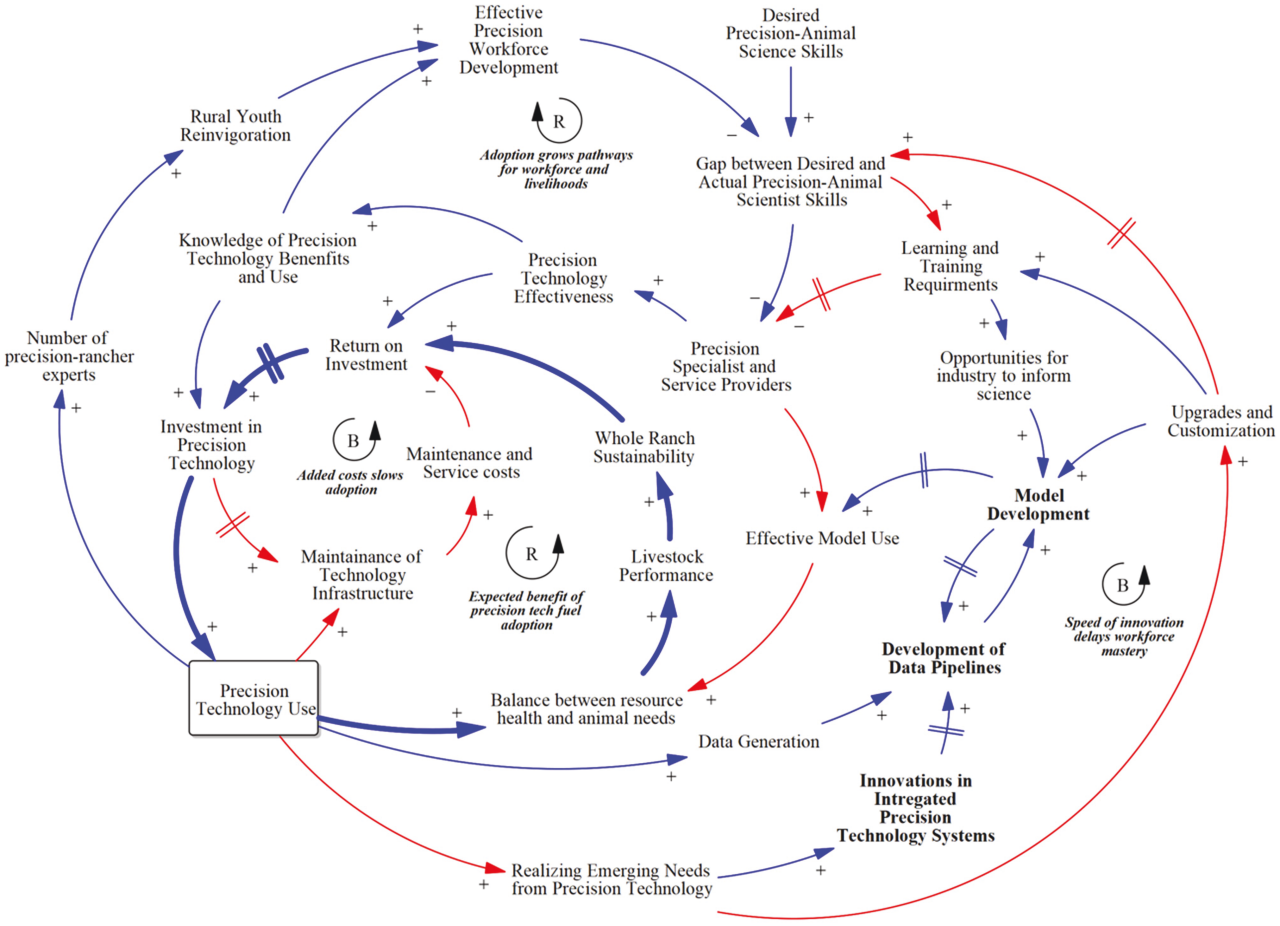
Causal loop diagram of precision livestock integration through precision system models, education workforce development, and producer and industry synergies. A positive (+) relationship between variables indicates that as the value of the arrowhead moves in the same direction (increases or decreases) as the variable at the tail (e.g., as Precision Technology Use increases, the Maintenance of Technology Infrastructure also increases). A negative (–) relationship between variables indicates that the variable at the arrowhead moves in the opposite direction of the variable at the tail (e.g., as Learning and Training Requirements increases, the number of Precision Specialist and Service Providers decreases). The double perpendicular lines on arrows represent time delays between variable responses. The R and B labels identify reinforcing (positive feedback) or balancing (negative feedback) relationships.

Maximizing the potential benefits of precision livestock production and PSM implementation requires the consideration of longer-term production capacity and human capital needs. Thus, a causal loop diagram was developed to illustrate the complexity of precision livestock production system development and adoption (Figure 5). Causal loop diagrams identify the complex feedback relationships (goal-seeking balancing relationships or reinforcing effects that either build or erode desired synergies) and associated time delays for key variables (Sterman, 2000) that would otherwise make understanding the effects of a change to a system difficult (e.g., shifting from conventional to precision livestock production). These feedback mechanisms drive the behavior (e.g., animal productivity) of a system and determine the level of response to changes in management decisions. Understanding which feedback mechanisms either drive or constrain precision livestock production capacity and implementation is critical to gain a more holistic understanding.

Improving system understanding through the long-term effectiveness in precision livestock systems requires producers, industry, and researcher entities to activate a reinforcing mechanism that enhances animal production and productivity, enhances economic profitability, and consequently fuels adoption (Figure 5). As knowledge, benefits, and implementation of precision technology expand, it will create a precision workforce of professionals and aspiring young adults in community colleges, technical colleges, and universities seeking to improve the agricultural industry through precision technology (Figure 5). An effective precision workforce will provide sufficient labor capacity, helping to keep maintenance and service costs well below the enhanced revenues gained from precision technology (Figure 5).

While there are numerous benefits to precision technology, there have been anecdotal observations that suggest hidden feedback processes reside below our collective surface of awareness. For example, as more precision technology is employed in real-world production environments, producers and consultants have greater opportunities to observe the emerging needs and prospects that precision technology can fill in confined and extensive systems. As this information is relayed back to technology developers, both hardware and software upgrades and customizations are made in an attempt to address these emergent gaps. The gap between desired and actual levels of precision animal science skills is widened, as it will take significant time for existing experts to incorporate new information into their knowledge-base and service capabilities. Therefore, it may be more challenging to produce the number of trained specialists and service providers needed as learning and training requirements increase (i.e., workforce mastery; Figure 5).

The way to overcome the unintended fragmentation of workforce mastery is through modeling and the scientific research process. By harnessing the expected exponential growth in quantitative data, the adjustments to current technology features, and the emergent technology capabilities under development, animal science researchers will be better equipped to conceptualize, parameterize, and test valid PSMs that meet the criteria of production systems to result in sustainable improvements as well as the clarity and user-flexibility needed to communicate model insights to managers. Lastly, a much-needed system improvement link will match industry stakeholders with scientists to provide more opportunities for those in the field, facilitating technology transfer and management support to inform researchers about producers’ goals, constraints, and habits that to-date have not been well integrated into individual precision applications. Incorporating human dimensions into model development will hedge against future unintended consequences of precision technology innovations and facilitate managers adopting and experimenting with such tools (Figure 1).

## Conclusion

Successful implementation of precision technology is critical for both confined and extensive systems. PSMs are required to achieve desired goals, and many opportunities exist to leverage current MM to achieve effective PSM. The five principles for sustainable precision livestock implementation are 1) determining a performance gap, 2) increasing data collection and analysis capabilities, 3) determining the optimal solution with aid of precision systems modeling, 4) informing and implementing management changes, and 5) measuring systems-level responses and information feedback to remaining performance gaps (Figure 2) create an outline for evaluating opportunities and overcoming implementation challenges of precision livestock production. Synergistic relationships between managers, industry (i.e., tech firms), and researchers will enable sustainable and long-term success while avoiding unintended consequences (Figure 5). Precision livestock production in extensive systems is likely to have a tremendous impact given the environmental scope (54% of global surface area) and livestock numbers represented within extensive production settings. Technological advances required for extensive systems are likely to help further refine confined systems because they operate with far less infrastructure. Finally, the standardization of precision livestock management in extensive and confined systems will lead to better communication across supply chains (Menendez and Tedeschi, 2020) and consequently enhance consumer perception of animal production, especially regarding quality, environmental sustainability, and welfare.

## Abbreviations

DLF: digital livestock farming
GHG: greenhouse gas
MM: mathematical modeling
PLF: precision livestock farming
PSM: precision system models

## References

[CIT0001] Abdelkrim, A., L. Puillet, P. Gomes, and O. Martin. 2021. Lactation curve model with explicit representation of perturbations as a phenotyping tool for dairy livestock precision farming. Animal. 15:100074. doi:10.1016/j.animal.2020.100074 33515999

[CIT0002] Aderinto, R. F., A. O. Anoruo, R. Machen, and B. L. Turner. 2020. Can the tragedy of the commons be avoided in common-pool forage resource systems? An application to small-holder herding in the semi-arid grazing lands of Nigeria. Sustainability. 12:5947. doi:10.3390/su12155947

[CIT0003] Ahrendt, P., T. Gregersen, and H. Karstof. 2011. Development of a real-time computer vision system for tracking loose-housed pigs. Comput. Electron. Agric. 76:169–174. doi:10.1016/j.compag.2010.10.013

[CIT0004] Alstrup, L., M. O. Nielsen, P. Lund, J. Sehested, M. K. Larsen, and M. R. Weisbjerg. 2015. Milk yield, feed efficiency and metabolic profiles in Jersey and Holstein cows assigned to different fat supplementation strategies. Livest. Sci. 178:165–176. doi:10.1016/j.animal.2021.100429

[CIT0005] Anderson, D. M., R. E. Estell, J. L. Holechek, S. Ivey, and G. B. Smith. 2014. Virtual herding for flexible livestock management – a review. Rangeland J. 36:205–221. doi:10.1071/RJ13092

[CIT0006] Andretta, I., C. Pomar, M. Kipper, L. Hauschild, and J. Rivest. 2016a. Feeding behavior of growing–finishing pigs reared under precision feeding strategies. J. Anim. Sci. 94:3042–3050. doi:10.2527/jas.2016-0392 27482691

[CIT0007] Andretta, I., C. Pomar, J. Rivest, J. Pomar, and J. Radünz. 2016b. Precision feeding can significantly reduce lysine intake and nitrogen excretion without compromising the performance of growing pigs. Animal. 10:1137–1147. doi:10.1017/S1751731115003067 26759074

[CIT0008] Aquilani, C., A. Confessore, R. Bozzi, F. Sirtori, and C. Pugliese. 2022. Review: Precision Livestock Farming technologies in pasture-based livestock systems. Animal. 16:100429. doi:10.1016/j.animal.2021.100429 34953277

[CIT0009] Arbel, R., Y. Bigun, E. Ezra, H. Sturman, and D. Hojman. 2001. The effect of extended calving intervals in high lactating cows on milk production and profitability. J. Dairy Sci. 84:600–608. doi:10.3168/jds.s0022-0302(01)74513-4 11286412

[CIT0010] Aubrey, A. 2019. This diet is better for the planet. But is it better for you, too? National Public Radio. Available from https://www.npr.org/sections/thesalt/2019/01/27/688765872/this-diet-is-better-for-the-planet-but-is-it-better-for-you-too. Accessed July 7, 2021.

[CIT0011] Augustine, D. J., and J. D. Derner. 2013. Assessing herbivore foraging behavior with GPS collars in a semiarid grassland. Sensors. 13:3711–3723. doi:10.3390/s130303711 23503296 PMC3658770

[CIT0012] Augustine, D. J., and J. D. Derner. 2014. Controls over the strength and timing of fire-grazer interactions in a semi-arid rangeland. J. Appl. Ecol. 51:242–250. doi:10.1111/1365-2664.12186

[CIT0013] Ausseil, A.-G., J. Dymond, R. Dynes, J. D. Shepherd, B. Vantier, and A. Sutherland. 2011. Estimating pasture quality using Landsat ETM+: application for the greenhouse gas inventory of New Zealand. Proceeding of the International Symposium on Remote Sensing for the Environment; Sydney, Australia.

[CIT0014] Bailey, D. W. 2005. Identification and creation of optimum habitat conditions for livestock. Rangeland Ecol. Manag. 58:109–118.

[CIT0015] Bailey, D. W., S. Lunt, A. Lipka, M. G. Thomas, J. F. Medrano, A. Canovas, G. Rincon, M. B. Stephenson, and D. Jensen. 2015. Genetic influences on cattle grazing distribution: association of genetic markers with terrain use in cattle. Rangeland Ecol. Manag. 68:142–149. doi:10.1016/j.rama.2015.02.001

[CIT0016] Bailey, D. W., M. Trotter, C. Tobin, and M. G. Thomas. 2021. Opportunities to apply precision livestock management on rangelands. Front. Sustain. Food Syst. 5:611915. doi:10.3389/fsufs.2021.611915

[CIT0017] Bailey, D. W., H. C. VanWagoner, R. Weinmeister, and D. Jensen. 2008. Evaluation of low-stress herding and supplement placement for managing cattle grazing in riparian and upland areas. Rangeland Ecol. Manag. 61:26–37. doi:10.2111/06-130.1

[CIT0018] Baumgard, L. H., and P. Rhodes, Jr. 2013. Effects of heat stress of postabsorptive metabolism and energetics. Ann. Rev. Anim. Biosci. 1:311–337. doi:10.1146/annurev-animal-031412-103644 25387022

[CIT0019] Benjamin, M., and S. Yik. 2019. Precision livestock farming in swine welfare: a review for swine practitioners. Animals. 9:133. doi:10.3390/ani9040133 PMC6523486 30935123

[CIT0020] Berckmans, D. 2017. General introduction to precision livestock farming. Anim. Front. 7:6–11. doi:10.2527/af.2017.0102

[CIT0021] Bernabucci, U. 2019. Climate change: impact on livestock and how can we adapt. Anim. Front. 9:3–5. doi:10.1093/af/vfy039 PMC6951910 32002232

[CIT0022] Bossen, D., and M. R. Weisbjerg. 2009. Allocation of feed based on individual dairy cow live weight changes II: effect on milk production. Livest. Sci 126:273–285. doi:10.1016/j.livsci.2009.07.011

[CIT0023] Bossen, D., M. R. Weisbjerg, L. Munksgaard, and S. Højsgaard. 2009. Allocation of feed based on individual dairy cow live weight changes: I: feed intake and live weight changes during lactation. Livest. Sci. 126:252–272. doi:10.1016/j.livsci.2009.07.010

[CIT0024] Bowen, M. K., P. M. Pepper, R. C. McPhie, and M. R. Winter. 2009. Evaluation of a remote drafting system for regulating sheep access to supplement. Anim. Prod. Sci. 49:248. doi:10.1071/EA08161

[CIT0025] Brennan, J. R., P. Johnson, and K. Olson. 2021. Classifying Season Long Livestock Grazing Behavior with the use of a low-cost GPS and accelerometer. Comput. Electron. Agric. 181:105957. doi:10.1016/j.compag.2020.105957

[CIT0026] Brossard, L., J. Y. Dourmad, J. Rivest, and J. van Milgen. 2009. Modelling the variation in performance of a population of growing pig as affected by lysine supply and feeding strategy. Animal. 3:1114–1123. doi:10.1017/S1751731109004546 22444841

[CIT0027] Brown-Brandl, T. M., F. Adrion, J. Maselyne, A. Kapun, E. F. Hessel, W. Saeys, A. Van Nuffel, and E. Gallmann. 2019. A review of passive radio frequency identification systems for animal monitoring in livestock facilities. Appl. Eng. Agric. 35:579–591. doi:10.13031/aea.12928

[CIT0028] Browning, D. M., A. Rango, J. W. Karl, C. M. Laney, E. R. Vivoni, and C. E. Tweedie. 2015. Emerging technological and cultural shifts advancing drylands research and management. Front. Ecol. Environ. 13:52–60. doi:10.1890/140161

[CIT0029] Butler, W. R., and R. D. Smith. 1989. Interrelationships between energy balance and postpartum reproductive function in dairy cattle. J. Dairy Sci. 72:767–783. doi:10.3168/jds.S0022-0302(89)79169-4 2654227

[CIT0030] Cabrera, V. E., and L. Fadul-Pacheco. 2021. Future of dairy farming from the Dairy Brain perspective: data integration, analytics, and applications. Int. Dairy J. 121:105069. doi:10.1016/j.idairyj.2021.105069

[CIT0031] Caja, G., A. Castro-Costa, A. A. K. Salama, J. Oliver, M. Baratta, C. Ferrer, and C. H. Knight. 2020. Sensing solutions for improving the performance, health and wellbeing of small ruminants. J. Dairy Res. 87:34–46. doi:10.1017/S0022029920000667 33213578

[CIT0032] Cang, Y., H. He, and Y. Qiao. 2019. An intelligent pig weights estimate method based on deep learning in sow stall environments. IEEE Access 7:164867–164875. doi:10.1109/ACCESS.2019.2953099

[CIT0033] Capper, J. L. 2011. The environmental impact of beef production in the United States: 1977 compared with 2007. J. Anim. Sci. 89:4249–4261. doi:10.2527/jas.2010-3784 21803973

[CIT0034] Cellier, M., C. Duvaux-Ponter, and B. L. Nielsen. 2021. Inter- and intra-individual variability of feeding behaviour in group housed dairy goats. Appl. Anim. Behav. Sci. 234:105167. doi:10.1016/j.applanim.2020.105167

[CIT0035] Chalak, M., and D. J. Pannell. 2012. Optimising control of an agricultural weed in sheep-production pastures. Agric. Syst. 109:1–8. doi:10.1016/j.agsy.2012.01.010

[CIT0036] Conant, R. T., J. Six, and K. Paustian. 2003. Land use effects on soil carbon fractions in the southeastern United States. I. Management-intensive versus extensive grazing. Biol. Fertil. Soils. 38:386–392. doi:10.1007/s00374-003-0652-z

[CIT0037] Cronin, M. A., C. Gonzalez, and J. D. Sterman. 2009. Why don’t well-educated adults understand accumulation? A challenge to researchers, educators, and citizens. Organ. Behav. Hum. Decis. Process 108:116–130. doi:10.1016/j.obhdp.2008.03.003

[CIT0038] Dourmad, J. Y., M. Etienne, A. Malancogne, S. Dubois, J. van Milgen, and J. Noblet. 2008. InraPorc: a model and decision support tool for the nutrition of sows. Anim. Feed Sci. Tech. 143:372–386. doi:10.1016/j.anifeedsci.2007.05.019

[CIT0039] Dumas, A., J. Dijkstra, and J. France. 2008. Mathematical modelling in animal nutrition: a centenary review. J. Agric. Sci. 146:123–142. doi:10.1017/S0021859608007703

[CIT0040] Ellis, J., M. Jacobs, J. Dijkstra, H. van Laar, J. Cant, D. Tulpan, and N. Ferguson. 2019. The role of mechanistic models in the era of big data and intelligent computing. Adv. Anim. Biosci. 10:286.

[CIT0041] Ellis, J. L., M. Jacobs, J. Dijkstra, H. van Laar, J. P. Cant, D. Tulpan, and N. Ferguson. 2020. Review: Synergy between mechanistic modelling and data-driven models for modern animal production systems in the era of big data. Animal. 14:s223–s237. doi:10.1017/S1751731120000312 32141423

[CIT0042] Erdman, R. A., and M. Varner. 1995. Fixed yield responses to increased milking frequency. J. Dairy Sci. 78:1199–1203. doi:10.3168/jds.S0022-0302(95)76738-8 7622729

[CIT0043] Fernandes, A. F. A., J. R. R. Dórea, R. Fitzgerald, W. Herring, and G. J. M. Rosa. 2019. A novel automated system to acquire biometric and morphological measurements and predict body weight of pigs via 3D computer vision. J. Anim. Sci. 97:496–508. doi:10.1093/jas/sky418 30371785 PMC6313152

[CIT0044] Fogarty, E. S., D. L. Swain, G. M. Cronin, L. E. Moraes, D. W. Bailey, and M. Trotter. 2021. Developing a simulated online model that integrates GNSS, accelerometer and weather data to detect parturition events in grazing sheep: a machine learning approach. Animal 11:303. doi:10.3390/ani11020303 PMC7911250 33503953

[CIT0045] Food and Agriculture Organization (FAO). 2011. World Livestock 2011 – livestock in food security. Rome (Italy): Food and Agriculture Organization.

[CIT0046] Food and Agriculture Organization (FAO). 2020. Livestock and environment statistics: manure and greenhouse gas emissions. Global, regional and country trends, 1990–2018. FAOSTAT Analytical Brief Series No. 14. Rome (Italy): Food and Agriculture Organization.

[CIT0047] Food and Agriculture Organization (FAO). 2021. Facts and Findings. Food and Agriculture Organization. Available from http://www.fao.org/news/story/en/item/197623/icode// [accessed December 17, 2021].

[CIT0048] Franke, J., V. Keuck, and F. Siegert. 2012. Assessment of grassland use intensity by remote sensing to support conservation schemes. J. Nat. Conserv. 20:125–134. doi:10.1016/j.jnc.2012.02.001

[CIT0049] Friggens, N. C., L. Brun-Lafleur, P. Faverdin, D. Sauvant, and O. Martin. 2013. Advances in predicting nutrient partitioning in the dairy cow: recognizing the central role of genotype and its expression through time. Animal. 7:89–101. doi:10.1017/S1751731111001820 23031683

[CIT0050] Fromm, J. 2019. Meat and the modern consumer. Forbes. Available from https://www.forbes.com/sites/jefffromm/2019/03/19/meat-and-the-modern-consumer/#650a2236343c. Accessed July 1, 2021.

[CIT0051] Fu, Q., W. Shen, X. Wei, Y. Zhang, H. Xin, Z. Su, and C. Zhao. 2020. Prediction of the diet energy digestion using kernel extreme learning machine: a case study with Holstein dry cows. Comput. Electron. Agric. 169:105231. doi:10.1016/j.compag.2020.105231

[CIT0052] Gaillard, C., L. Brossard, and J. Y. Dourmad. 2020a. Review – Improvement of feed and nutrient efficiency in pig production through precision feeding. Anim. Feed Sci. Tech. 268:114611. doi:10.1016/j.anifeedsci.2020.114611

[CIT0053] Gaillard, C., R. Gauthier, L. Cloutier, and J. Y. Dourmad. 2019. Exploration of individual variability to better predict the nutrient requirements of gestating sows. J. Anim. Sci. 97:4934–4945. doi:10.1093/jas/skz320 31608374 PMC6915206

[CIT0054] Gaillard, C., O. Martin, P. Blavy, N. Friggens, J. Sehested, and H. Phuong. 2016. Prediction of the reproductive lifetime performance of Holstein cows managed for different durations, using a model of lifetime nutrient partitioning. J. Dairy Sci. 99:9126–9135. doi:10.3168/jds.2016-11051 27568052

[CIT0055] Gaillard, C., N. Quiniou, R. Gauthier, L. Cloutier, and J. Y. Dourmad. 2020b. Evaluation of a decision support system for precision feeding of gestating sows. J. Anim. Sci. 98:1–12. doi:10.1093/jas/skaa255 PMC7568449 32776149

[CIT0056] García, S. C., and W. J. Fulkerson. 2005. Opportunities for future Australian dairy systems: a review. Aust. J. Exp. Agric. 45:1041–1055. doi:10.1071/EA04143

[CIT0057] Gauthier, R., C. Largouët, C. Gaillard, L. Cloutier, F. Guay, and J. Y. Dourmad. 2019. Dynamic modeling of nutrient use and individual requirements of lactating sows. J. Anim. Sci. 97:2822–2836. doi:10.1093/jas/skz167 31115459 PMC6606508

[CIT0058] Gerber, P. J., H. Steinfeld, B. Henderson, A. Mottet, C. Opio, J. Dijkman, A. Falcucci, and G. Tempio. 2013. Tackling climate change through livestock – a global assessment of emissions and mitigation opportunities. Rome (Italy): Food and Agriculture Organization of the United Nations (FAO).

[CIT0059] Giger-Reverdin, S., C. Duvaux-Ponter, D. Sauvant, and N. C. Friggens. 2020. Repeatability of traits for characterizing feed intake patterns in dairy goats: a basis for phenotyping in the precision farming context. Animal. 14:1083–1092. doi:10.1017/S1751731119002817 31769385 PMC7163394

[CIT0060] Gomez, A., and N. B. Cook. 2010. Time budgets of lactating dairy cattle in commercial freestall herds. J. Dairy Sci. 93:5772–5781. doi:10.3168/jds.2010-3436 21094749

[CIT0061] Goodin, D. G., and G. M. Henebry. 1997. A technique for monitoring ecological disturbance in tallgrass prairie using seasonal NDVI trajectories and a discriminant function mixture model. Remote Sens. Environ. 61:270–278. doi:10.1016/S0034-4257(97)00043-6

[CIT0062] Groher, T., K. Heitkämper, and C. Umstätter. 2020. Digital technology adoption in livestock production with a special focus on ruminant farming. Animal. 14:2404–2413. doi:10.1017/S1751731120001391 32613933 PMC7538341

[CIT0063] Grossi, G., P. Goglio, A. Vitali, and A. G. Williams. 2019. Livestock and climate change: impact of livestock on climate and mitigation strategies. Anim. Front. 9(1):69–76. doi:10.1093/af/vfy034 PMC7015462 32071797

[CIT0064] Halachmi, I., M. Guarino, J. Bewley, and M. Pastell. 2019. Smart animal agriculture: application of real-time sensors to improve animal well-being and production. Annu. Rev. Anim. Biosci. 7:403–425. doi:10.1146/annurev-animal-020518-114851 30485756

[CIT0065] Hansen, T. L., M. Li, J. Li, C. J. Vankerhove, M. A. Sotirova, J. M. Tricarico, V. E. Cabrera, E. Kebreab, and K. F. Reed. 2021. The ruminant farm systems animal module: a biophysical description of animal management. Animals (Basel). 11:1373. doi:10.3390/ani11051373 34066009 PMC8151839

[CIT0066] Hauschild, L., A. Kristensen, I. Andretta, A. Remus, L. Santos, and C. Pomar. 2020. Toward better estimates of the real-time individual amino acid requirements of growing-finishing pigs showing deviations from their typical feeding patterns. Animal. 14:s371–s381. doi:10.1017/S1751731120001226 32515319

[CIT0067] Hauschild, L., P. A. Lovatto, J. Pomar, and C. Pomar. 2012. Development of sustainable precision farming systems for swine: estimating real-time individual amino acid requirements in growing-finishing pigs. J. Anim. Sci. 90:2255–2263. doi:10.2527/jas.2011-4252 22287679

[CIT0068] Helwatkar, A., D. Riordan, and J. Walsh. 2014. Sensor technology for animal health monitoring. Proceedings of the 8th International Conference on Sensing Technology. Liverpool (UK): World Academy of Science, Engineering, and Technology; p. 266–271.

[CIT0069] Intergovernmental Panel on Climate Change (IPCC). 2019. Climate change and land. In: Shukla, P. R., J. Skea, E. Calvo Buendia, V. Masson-Delmotte, H.O. Pörtner, D.C. Roberts, P. Zhai, R. Slade, S. Connors, R. van Diemen, et al., editors. An IPCC special report on climate change, desertification, land degradation, sustainable land management, food security, and greenhouse gas fluxes in terrestrial ecosystems. Geneva (Switzerland): IPCC.

[CIT0070] Jacobs, E. C., A. Remus, C. Gaillard, H. M. Menendez III, L. O. Tedeschi, S. Neethirajan and J. L. Ellis. 2022. ASAS-NANP SYMPOSIUM: Limitations and potential next steps for modeling and modelers in the Animal Sciences. J. Anim. Sci. (Companion Paper). doi:10.1093/jas/skac132 PMC9171330 35419602

[CIT0071] Jensen, C. 2014. Milk and growth responses to energy intake in dairy cattle – in the perspective of the non-additive feed evaluation system – NorFor [PhD thesis]. Aarhus (DK): Science and Technology, Aarhus University.

[CIT0072] Jones, M. O., N. P. Robinson, D. E. Naugle, J. D. Maestas, M. C. Reeves, R. W. Lankston, and B. W. Allred. 2021. Annual and 16-day rangeland production estimates for the western United States. Rangeland Ecol. Manag. 77:112–117. doi:10.1016/j.rama.2021.04.003

[CIT0073] Jordan, D. J., S. Hatcher, G. J. Lee, I. McConnel, M. Bowen, A. J. Bosca, and J. B. Rowe. 2006. Nutritional management for reproductive efficiency. Int. J. Sheep Wool Sci. 54:35–41.

[CIT0074] Jouven, M., H. Leroy, A. Ickowicz, and P. Lapeyronie. 2012. Can virtual fences be used to control grazing sheep?. Rangeland J. 34:111–123. doi:10.1071/RJ11044

[CIT0075] Kaler, J., and A. Ruston. 2019. Technology adoption on farms: using Normalisation Process Theory to understand sheep farmers’ attitudes and behaviours in relation to using precision technology in flock management. Prev. Vet. Med. 170:104715. doi:10.1016/j.prevetmed.2019.104715 31421497 PMC6745618

[CIT0076] Karl, J. W., and J. E. Sprinkle. 2019. Low-cost livestock global positioning system collar from commercial off-the-shelf parts. Rangeland Ecol. Manag. 72:954–958. doi:10.1016/j.rama.2019.08.003

[CIT0077] Kashiha, M., A. Pluk, C. Bahr, E. Vranken, and D. Berckmans. 2013. Development of an early warning system for a broiler house using computer vision. Biosyst. Eng. 116:36–45. doi:10.1016/j.biosystemseng.2013.06.004

[CIT0078] Kebreab, E., K. F. Reed, V. E. Cabrera, P. A. Vadas, G. Thoma, and J. M. Tricarico. 2019. A new modeling environment for integrated dairy system management. Anim. Front. 9:25–32. doi:10.1093/af/vfz004 32002248 PMC6951933

[CIT0079] Klei, L. R., J. M. Lynch, D. M. Barbano, P. A. Oltenacu, A. J. Lednor, and D. K. Bandler. 1997. Influence of milking three times a day on milk quality. J. Dairy Sci. 80:427–436. doi:10.3168/jds.S0022-0302(97)75954-X 9098793

[CIT0080] Klerkx, L., E. Jakku, and P. Labarthe. 2019. A review of social science on digital agriculture, smart farming and agriculture 4.0: new contributions and a future research agenda. Njas-Wagen. J. Life Sci. 90:100315. doi:10.1016/j.njas.2019.100315

[CIT0081] Knight, C. H. 2005. Extended lactation: turning theory into reality. Adv. Dairy Technol. 17:113–123.

[CIT0082] Knight, C. W., D. W. Bailey, and D. Faulkner. 2018. Low-cost global positioning system tracking collars for use on cattle. Rangeland Ecol. Manag. 71:506–508. doi:10.1016/j.rama.2018.04.003

[CIT0083] Kolver, E. S., and L. D. Muller. 1998. Performance and nutrient intake of high producing Holstein cows consuming pasture or a total mixed ration. J. Dairy Sci. 81:1403–1411. doi:10.3168/jds.S0022-0302(98)75704-2 9621244

[CIT0084] Kumar, S., H. Sieverding, L. Lai, N. Thandiwe, B. Wienhold, D. Redfearn, D. Archer, D. Ussiri, D. Faust, D. Landblom, et al. 2019. Facilitating crop–livestock reintegration in the Northern Great Plains. Agron. J. 111:2141–2156. doi:10.2134/agronj2018.07.0441

[CIT0085] Labrecque, J., F. Gouineau, J. Rivest, and G. Germain. 2020. Real-time tracking of individual pigs and collection of behavioral metrics using security cameras. In: O’Brien B., D. Hennessy, and L. Shallo, editors, Precision livestock farming ‘19. The Organising Committee of the 9th European Conference on Precision Livestock Farming (ECPLF), Teagasc. Moorepark Co. Cork, Fermoy, IE: Animal & Grassland Research and Innovation Centre; p. 460–466.

[CIT0086] Machado, S. C., C. M. McManus, M. T. Stumpf, and V. Fischer. 2014. Concentrate: forage ratio in the diet of dairy cows does not alter milk physical attributes. Trop. Anim. Health Prod. 46:855–859. doi:10.1007/s11250-014-0576-7 24647476

[CIT0087] Maltz, E., L. F. Barbosa, P. Bueno, L. Scagion, K. Kaniyamattam, L. F. Greco, A. De Vries, and J. E. Santos. 2013. Effect of feeding according to energy balance on performance, nutrient excretion, and feeding behavior of early lactation dairy cows. J. Dairy Sci. 96:5249–5266. doi:10.3168/jds.2013-6549 23726421

[CIT0088] Mansbridge, N., J. Mitsch, N. Bollard, K. Ellis, G. G. Miguel-Pacheco, T. Dottorini, and J. Kaler. 2018. Feature selection and comparison of machine learning algorithms in classification of grazing and rumination behavior in sheep. Sensors. 18:3532. doi:10.3390/s18103532 PMC6210268 30347653

[CIT0089] Marini, D., F. Cowley, S. Belson, and C. Lee. 2019. The importance of an audio cue warning in training sheep to a virtual fence and differences in learning when tested individually or in small groups. Appl. Anim. Behav. Sci. 221:104862. doi:10.1016/j.applanim.2019.104862

[CIT0090] Marini, D., T. Kearton, J. Ouzman, R. Llewellyn, S. Belson, and C. Lee. 2020. Social influence on the effectiveness of virtual fencing in sheep. PeerJ. 8:e10066. doi:10.7717/peerj.10066 33062448 PMC7532778

[CIT0091] Marini, D., R. Llewellyn, S. Belson, and C. Lee. 2018. Controlling within-field sheep movement using virtual fencing. Animals (Basel). 8:31. doi:10.3390/ani8030031 PMC5867519 29495364

[CIT0092] Martin, O., and D. Sauvant. 2010. A teleonomic model describing performance (body, milk and intake) during growth and over repeated reproductive cycles throughout the lifespan of dairy cattle. 1. Trajectories of life function priorities and genetic scaling. Animal. 4:2030–2047. doi:10.1017/S1751731110001357 22445378

[CIT0093] McGranahan, D., B. Geaumont, and J. W. Spiess. 2018. Assessment of a livestock GPS collar based on an open-source datalogger informs best practices for logging intensity. Ecol. Evol. 8:5649–5660. doi:10.1002/ece3.4094 29938081 PMC6010917

[CIT0094] Menendez, H. M., and L. O. Tedeschi. 2020. The characterization of the cow-calf, stocker and feedlot cattle industry water footprint to assess the impact of livestock water use sustainability. J. Agric. Sci. 158:416–430. doi:10.1017/S0021859620000672

[CIT0095] Morota, G., R. V. Ventura, F. F. Silva, M. Koyama, and S. C. Fernando. 2018. Big data analytics and precision animal agriculture symposium: machine learning and data mining advance predictive big data analysis in precision animal agriculture. J. Anim. Sci. 96:1540–1550. doi:10.1093/jas/sky014 29385611 PMC6140937

[CIT0096] National Academies of Sciences, Engineering, and Medicine (NASEM). 2016. Nutrient requirements of beef cattle. 8th rev. ed. Washington, DC: National Academies Press.

[CIT0097] National Aeronautics and Space Administration (NASA). 2021. Earth data. Available from https://earthdata.nasa.gov/. [accessed December 10, 2021].

[CIT0098] National Research Council (NRC). 2007. Nutrient requirements of small ruminants: sheep, goats, cervids, and new world camelids. Washington, DC: National Academies Press.

[CIT0099] National Resources Conservation Service (NRCS). 2021. Web soil survey. Available from https://websoilsurvey.sc.egov.usda.gov/App/HomePage.htm [accessed December 5, 2021].

[CIT0100] Navon, S., A. Mizrach, A. Hetzroni, and E. D. Ungar. 2013. Automatic recognition of jaw movements in free-ranging cattle, goats and sheep, using acoustic monitoring. Biosystems Eng. 114: 474–483. doi:10.1016/j.biosystemseng.2012.08.005

[CIT0101] Neethirajan, S. 2017. Recent advances in wearable sensors for animal health management. Sens. Bio-Sens. Res. 12:15–29. doi:10.1016/j.sbsr.2016.11.004

[CIT0102] Neethirajan, S. 2021a. The use of artificial intelligence in assessing affective states in livestock. Front. Vet. Sci. 8:1–8. doi:10.3389/fvets.2021.715261 PMC8364945 34409091

[CIT0103] Neethirajan, S. 2021b. ChickTrack – a quantitative tracking tool for measuring chicken activity. TechRxiv, doi:10.36227/techrxiv.15031440.v1, July 26, 2021, preprint: not peer reviewed.

[CIT0104] Neethirajan S. 2021c. Happy cow or thinking pig? Wur wolf—facial coding platform for measuring emotions in farm animals. AI. 2(3):342–354. doi:10.3390/ai2030021

[CIT0105] Neethirajan, S., and B. Kemp. 2021a. Digital livestock farming. Sens. Bio-Sens. Res. 32:100408. doi:10.1016/j.sbsr.2021.100408

[CIT0106] Neethirajan, S., and B. Kemp. 2021b. Digital phenotyping in livestock farming. Animals. 11:2009. doi:10.3390/ani11072009 34359137 PMC8300347

[CIT0107] Neethirajan, S., K. V. Ragavan, and X. Weng. 2018. Agro-defense: biosensors for food from healthy crops and animals. Trends Food Sci. Technol. 73:25–44. doi:10.1016/j.tifs.2017.12.005

[CIT0108] Norton, T., C. Chen, M. L. V. Larsen, and D. Berckmans. 2019. Precision livestock farming: building ‘digital representations’ to bring the animals closer to the farmer. Animal. 13(12):3009–3017. doi:10.1017/S175173111900199X 31516101

[CIT0109] Oates, L. G., D. J. Undersander, C. Gratton, M. M. Bell, and R. D. Jackson. 2011. Management-intensive rotational grazing enhances forage production and quality of subhumid cool-season pastures. Crop Sci. 51:892–901. doi:10.2135/cropsci2010.04.0216

[CIT0110] Osterman, S., and J. Bertilsson. 2003. Extended calving interval in combination with milking two or three times per day: effects on milk production and milk composition. Livest. Prod. Sci. 82:139–149. doi:10.1016/S0301-6226(03)00036-8

[CIT0111] Otgonbayar, M., C. Atzberger, J. Chambers, and A. Damdinsuren. 2019. Mapping pasture biomass in Mongolia using partial least squares, random forest regression and Landsat 8 imagery. Int. J. Remote Sens. 40:3204–3226. doi:10.1080/01431161.2018.1541110

[CIT0112] Park, J. Y., S. Ale, W. R. Teague, and J. Jeong. 2017. Evaluating the ranch and watershed scale impacts of using traditional and adaptive multi-paddock grazing on runoff, sediment and nutrient losses in North Texas, USA. Agric. Ecosyst. Environ. 240:32–44. doi:10.1016/j.agee.2017.02.004

[CIT0113] Parsons, D. J., D. M. Green, C. P. Schofield, and C. T. Whittemore. 2007. Real-time control of pig growth through an integrated management system. Biosyst. Eng. 96:257–266. doi:10.1016/j.biosystemseng.2006.10.013

[CIT0114] Pearson, R. E., L. A. Fulton, P. D. Thompson, and J. W. Smith. 1979. Three times a day milking during the first half of the lactation. J. Dairy Sci. 62:1941–1950. doi:10.3168/jds.S0022-0302(79)83526-2 541464

[CIT0115] Peña Fernández, A., T. G. M. Demmers, Q. Tong, A. Youssef, T. Norton, E. Vranken, and D. Berckmans. 2019. Real-time modelling of indoor particulate matter concentration in poultry houses using broiler activity and ventilation rate. Biosyst. Eng. 187:214–225. doi:10.1016/j.biosystemseng.2019.09.004

[CIT0116] Phillips, R., O. Beeri, E. Scholljegerdes, D. Bjergaard, and J. Hendrickson. 2009. Integration of geospatial and cattle nutrition information to estimate paddock grazing capacity in Northern US prairie. Agric. Syst. 100:72–79. doi:10.1016/j.agsy.2009.01.002

[CIT0117] Phuong, H. N., O. Martin, I. M. J. de Boer, K. L. Ingvartsen, P. Schmidely, and N. C. Friggens. 2015. Deriving estimates of individual variability in genetic potentials of performance traits for 3 dairy breeds, using a model of lifetime nutrient partitioning. J. Dairy Sci. 98:618–632. doi:10.3168/jds.2014-8250 25465536

[CIT0118] Piñeiro, C., J. Morales, M. Rodríguez, M. Aparicio, E. G. Manzanilla, and Y. Koketsu. 2019. Big (pig) data and the internet of the swine things: a new paradigm in the industry. Anim. Front. 9:6–15. doi:10.1093/af/vfz002 PMC6951909 32002246

[CIT0119] Place, S. E., and F. M. Mitloehner. 2012. Beef production in balance: considerations for life cycle analysis. Meat Sci. 92:179–181. doi:10.1016/j.meatsci.2012.04.013 22551868

[CIT0120] Pomar, C., J. van Milgen, and A. Remus. 2019. Precision livestock feeding, principle and practice, poultry and pig nutrition. In: Hendriks, W.H., M.W.A. Verstegen, and L. Babainszky, editors. Poultry and pig nutrition: challenges of the 21st century. Wageningen, NL: Wageningen Academic Publishers; p. 397–418.

[CIT0121] Pomar, C., J. Pomar, J. Rivest, L. Cloutier, M. P. Letourneau-Montminy, I. Andretta, and L. Hauschild. 2015. Estimating real-time individual amino acid requirements in growing-finishing. In: Sakomura, N. K., R. M. Gous, I. Kyriazakis, and L. Hauschild, editors. Nutritional Modelling for Pigs and Poultry. Wallingford (UK): CABI Publishing; p. 157–174.

[CIT0122] Pomar, C., and A. Remus. 2019a. Are actual animal growth models adequate to predict growth and estimate amino acid and other nutrient requirements? In: Teixeira, I. A. M. A., B. Biagioli, L. Hauschild, and N. Sakomura, editors. Proceedings of the 9th Workshop on Modelling Nutrient Digestion and Utilization in Farm Animals (MODNUT) No. 10. Itamambuca (Brazil): Cambridge Universty Press; p. 294.

[CIT0123] Pomar, C., and A. Remus. 2019b. Precision pig feeding: a breakthrough toward sustainability. Anim. Front. 9:52–59. doi:10.1093/af/vfz006 PMC6951959 32002251

[CIT0124] Primi, R., G. Filibeck, A. Amici, C. Buckle, L. Cancellieri, A. Di Filippo, C. Gentile, A. Guglielmino, R. Latini, L. D. Mancini, et al. 2016. From Landsat to leafhoppers: a multidisciplinary approach for sustainable stocking assessment and ecological monitoring in mountain grasslands. Agric. Ecosyst. Environ. 234:118–133. doi:10.1016/j.agee.2016.04.028

[CIT0125] Pryce, J. E., M. D. Royal, P. C. Garnsworthy, and I. L. Mao. 2004. Fertility in the high-producing dairy cow. Livest. Prod. Sci. 86:125–135. doi:10.1016/S0301-6226(03)00145-3

[CIT0126] Puillet, L., and O. Martin. 2017. A dynamic model as a tool to describe the variability of lifetime body weight trajectories in livestock females. J. Anim. Sci. 95:4846–4856. doi:10.2527/jas2017.1803 29293698 PMC6292268

[CIT0127] Ramoelo, A., M. A. Cho, R. Mathieu, S. Madonsela, R. van de Kerchove, Z. Kaszta, and E. Wolff. 2015. Monitoring grass nutrients and biomass as indicators of rangeland quality and quantity using random forest modelling and World View-2 data. Int. J Appl. Earth Obs. Geoinf. 43:43–54. doi:10.1016/j.jag.2014.12.010

[CIT0128] Rangelands Atlas. 2021. Rangelands ATLAS. Available from https://www.rangelandsdata.org/atlas/sites/default/files/2021-06/Rangelands_web%20%28144%20dpi%29.pdf. [accessed December 10, 2021].

[CIT0129] Rao, Y., M. Jiang, W. Wang, W. Zhang, and R. Wang. 2020. On-farm welfare monitoring system for goats based on Internet of Things and machine learning. Int. J. Distrib. Sens. Netw. 16:1–17. doi:10.1177/1550147720944030

[CIT0130] Raynor, E. J., S. P. Gersie, M. B. Stephenson, P. E. Clark, S. A. Spiegal, R. K. Boughton, D. W. Bailey, A. Cibils, D. W. Smith, J. D. Derner, et al. 2021. Cattle grazing distribution related to topography across diverse rangeland ecosystems of North America. Rangeland Ecol. Manag. 75:91–103. doi:10.1016/j.rama.2020.12.002

[CIT0131] dos Reis, B. R., Z. Easton, R. R. White, and D. Fuka. 2021. A LoRa sensor network for monitoring pastured livestock location and activity. Transl. Anim. Sci. 5:1–9. doi:10.1093/tas/txab010 PMC8139410 34041440

[CIT0132] Remus, A., J. R. E. del Castillo, and C. Pomar. 2020a. Improving the estimation of amino acid requirements to maximize nitrogen retention in precision feeding for growing-finishing pigs. Animal. 14:2032–2041. doi:10.1017/S1751731120000798 32319362

[CIT0133] Remus, A., L. Hauschild, M. -P. Létourneau-Montminy, I. Andretta, and C. Pomar. 2020b. Feeding behavior of growing and finishing pigs fed different dietary threonine levels in a group-phase feeding and individual precision feeding system. Transl. Anim. Sci. 4:1–12. doi:10.1093/tas/txaa177 33196014 PMC7648131

[CIT0134] Remus, A., L. Hauschild, S. Methot, and C. Pomar. 2020c. Precision livestock farming: real-time estimation of daily protein deposition in growing–finishing pigs. Animal 14:s360–s370. doi:10.1017/S1751731120001469 32583758

[CIT0135] Ringgenberg, N., R. Bergeron, and N. Devillers. 2010. Validation of accelerometers to automatically record sow postures and stepping behaviour. Appl. Anim. Behav. Sci. 128:37–44. doi:10.1016/j.applanim.2010.09.018

[CIT0136] Robinson, T. P., P. K. Thornton, G. Franceschini, R. L. Kruska, F. Chiozza, A. Notenbaert, G. Cecchi, M. Herrero, M., Epprecht, S. Fritz, et al. 2011. Global livestock production systems. Rome, Italy: Food and Agriculture Organization of the United Nations (FAO) and International Livestock Research Institute (ILRI); pp. 1–145.

[CIT0137] Rojas-Downing, M., A. Pouran Nejadhashemi, T. Jarrigan, and S. A. Woznicki. 2017. Climate change and livestock: impacts, adaptation, and mitigation. Clim. Risk Manag. 16:145–163. doi:10.1016/j.crm.2017.02.001

[CIT0138] Roth, Z. 2015. Physiology and Endocrinology Symposium: Cellular and molecular mechanisms of heat stress related to bovine ovarian function. J. Anim. Sci. 93:2034–2044. doi:10.2527/jas.2014-8625 26020299

[CIT0139] Rotz, C. A., A. S. Asem-Hiablie, S. Place, and G. Thoma. 2019. Environmental footprints of beef cattle production in the United States. Agric. Syst. 169:1–13. doi:10.1016/j.agsy.2018.11.005

[CIT0140] Rowe, E., M. S. Dawkins, and S. G. Gebhardt-Henrich. 2019. A systematic review of precision livestock farming in the poultry sector: is technology focused on improving bird welfare? Animals (Basel). 9:614. doi:10.3390/ani9090614 PMC6770384 31461984

[CIT0141] Samperio, E., I. Lidón, R. Rebollar, M. Castejón-Limas, and C. Álvarez-Aparicio. 2021. Lambs’ live weight estimation using 3D images. Animal .15:100212. doi:10.1016/j.animal.2021.100212 34029788

[CIT0142] Sanderson, M. A., L. W. Jolley, and J. P. Dobrowolski. 2012. Pastureland and hayland in the USA: land resources, conservation practices, and ecosystem services. In: Nelson, C. J., editor. Conservation outcomes form pastureland and hayland practices: assessment, recommendations, and knowledge gaps. Lawrence (KS): Allen Press; p. 25–40.

[CIT0143] Sendra, S., F. Llario, L. Parra, and J. Lloret. 2013. Smart wireless sensor network to detect and protect sheep and goats to wolf attacks. Recent Adv. Comm. Netw. Tech. 2:91–101. doi:10.2174/22117407112016660012

[CIT0144] Smith, J. W., L. O. Ely, W. M. Graves, and W. D. Gilson. 2002. Effect of milking frequency on DHI performance measures. J. Dairy Sci. 85:3526–3533. doi:10.3168/jds.S0022-0302(02)74442-1 12512627

[CIT0145] Sprinkle, J. E., M. J. Ellison, J. B. Hall, J. V. Yelich, C. M. Willmore, and J. R. Brennan. 2021a. Grazing behavior and production for lactating cows differing in residual feed intake while grazing spring and summer rangeland. Transl. Anim. Sci. 5:1–23. doi:10.1093/tas/txab063 PMC8212170 34159296

[CIT0146] Sprinkle, J. E., J. K. Sagers, J. B. Hall, M. J. Ellison, J. V. Yelich, J. R. Brennan, T. B. Taylor, and J. B. Lamb. 2021b. Protein supplementation and grazing behavior for cows on differing late-season rangeland grazing systems. Animals. 11:3219. doi:10.3390/ani11113219 34827951 PMC8614474

[CIT0147] Stacey, K. F., D. J. Parsons, A. R. Frost, C. Fisher, D. Filmer, and A. Fothergill. 2004. An automatic growth and nutrition control system for broiler production. Biosyst. Eng. 89:363–371. doi:10.1016/j.biosystemseng.2004.07.006

[CIT0148] Stephens, E. C. 2021. ASAS-NANP Symposium: Review of systems thinking concepts and their potential value in animal science research. J. Anim. Sci. 99:1–7. doi:10.1093/jas/skab021 PMC8631063 33626146

[CIT0149] Stephenson, M. B., D. W. Bailey, R. A. Bruegger, and L. D. Howery. 2017. Factors affecting the efficacy of low-stress herding and supplement placement to target cattle grazing locations. Rangeland Ecol. Manag. 70:202–209. doi:10.1016/j.rama.2016.08.007

[CIT0150] Sterman, J. D. 1994. Learning in and about complex systems. Syst. Dynam. Rev. 10:291–330. doi:10.1002/sdr.4260100214

[CIT0151] Sterman, J. 2000. Business dynamics. New Delhi, IN: McGraw-Hill, Inc.

[CIT0152] Su, Q., J. Tang, M. Zhai, and D. He. 2022. An intelligent method for dairy goat tracking based on Siamese network. Comput. Electron. Agric. 193:106636. doi:10.1016/j.compag.2021.106636

[CIT0153] Taylor, J. K., R. L. Stanko, R. Rhoades, K. C. McCuistion, C. Mathis, R. Machen, and B. L. Turner. 2022. Can early weaning calves of first-calf heifers improve long-term herd and financial performance in a vertically integrated beef production system? A case-study application using system dynamics. Appl. Anim. Sci. 183–199. doi:10.15232/aas.2021-02235 (in press)

[CIT0154] Teague, R., and M. Barnes. 2017. Grazing management that regenerates ecosystem function and grazingland livelihoods. Afr. J. Range For. Sci. 34:77–86. doi:10.2989/10220119.2017.1334706

[CIT0155] Tedeschi, L. O. 2006. Assessment of the adequacy of mathematical models. Agric. Syst. 89:225–247. doi:10.1016/j.agsy.2005.11.004

[CIT0156] Tedeschi, L. O. 2019. ASN-ASAS Symposium: Future of Data Analytics in Nutrition: Mathematical modeling in ruminant nutrition: approaches and paradigms, extant models, and thoughts for upcoming predictive analytics. J. Anim. Sci. 97:1921–1944. doi:10.1093/jas/skz092 30882142 PMC6488328

[CIT0157] Tedeschi, L. O., M. A. Fonseca, J. P. Muir, D. P. Poppi, G. E. Carstens, J. P. Angerer, and D. G. Fox. 2017a. A glimpse of the future in animal nutrition science. 2. Current and future solutions. R. Bras. Zootec. 46:452–469. doi:10.1590/S1806-92902017000500012

[CIT0158] Tedeschi, L. O., and D. G. Fox. 2020. Ruminant nutrition system. Acton, MA: Xanedu Publishing Inc.

[CIT0159] Tedeschi, L. O., P. L., Greenwood, and I. Halachmi. 2021. Advancements in sensor technology and decision support intelligent tools to assist smart livestock farming. J. Anim. Sci. 99:1–11. doi:10.1093/jas/skab038 PMC7896629 33550395

[CIT0160] Tedeschi, L. O., A. Katiane de Almeida, A. S. Atzori, J. P. Muir, M. A. Fonseca, and A. Cannas. 2017b. A glimpse of the future in animal nutrition science. 1. Past and future challenges. R. Bras. Zootec. 46:438–451. doi:10.1590/S1806-92902017000500011

[CIT0161] Tedeschi, L. O., and H. M. Menendez III. 2020. Mathematical modeling in animal production. In: Bazer, F., G. C. Lamb, and G. Wu, editors. Animal agriculture. Cambridge, MA: Academic Press; p. 431–453.

[CIT0162] Tedeschi, L. O., G. Molle, H. M. Menendez, A. Cannas, and M. A. Fonseca. 2019. The assessment of supplementation requirements of grazing ruminants using nutrition models. Transl. Anim. Sci. 3:811–828. doi:10.1093/tas/txy140 32704848 PMC7250316

[CIT0163] Thornley, J. H., and J. France. 2007. Mathematical models in agriculture: quantitative methods for the plant, animal and ecological sciences. Oxfordshire, UK: CAB International.

[CIT0164] Thornton, P. K. 2010. Livestock production: recent trends, future prospects, Philos. Trans. R. Soc. B. 365:2853–2867. doi:10.1098/rstb.2010.0134 PMC2935116 20713389

[CIT0165] Tinsley, T. L., S. Chumbley, C. Mathis, R. Machen, and B. L. Turner. 2019. Managing cow herd dynamics in environments of limited forage productivity and livestock marketing channels: an application to semi-arid Pacific island beef production using system dynamics. Agric. Syst. 173:78–93. doi:10.1016/j.agsy.2019.02.014

[CIT0166] Tobin, C., D. W. Bailey, and M. G. Trotter. 2021. Tracking and sensor-based detection of livestock water system failure: a case study simulation. Rangeland Ecol. Manag. 77:9–16. doi:10.1016/j.rama.2021.02.013

[CIT0167] Tobin, C., D. Bailey, M. G. Trotter, and L. OʹConnor. 2020. Sensor based disease detection: a case study using accelerometers to recognize symptoms of bovine ephemeral fever. Comput. Electron. Agr. 175:105605. doi:10.1016/j.compag.2020.105605

[CIT0168] Todd, S. W., R. M. Hoffer, and D. G. Milchunas. 1998. Biomass estimation on grazed and ungrazed rangelands using spectral indices. Int. J. Remote Sens. 19:427–438. doi:10.1080/014311698216071

[CIT0169] Turner, B. L. 2020. Model laboratories: a quick-start guide for design of simulation experiments for dynamic systems models. Ecol. Model. 434:109246. doi:10.1016/j.ecolmodel.2020.109246

[CIT0170] Turner, B. L., M. Goodman, R. Machen, C. Mathis, R. Rhoades, and B. Dunn. 2020. Results of beer game trials played by natural resource managers versus students: does age influence ordering decisions? Systems 8:37. doi:10.3390/systems8040037

[CIT0171] Turner, B. L., H. M. Menendez, R. Gates, L. O. Tedeschi, and A. S. Atzori. 2016. System dynamics modeling for agricultural and natural resource management issues: review of some past cases and forecasting future roles. Resources. 540:1–24. doi:10.3390/resources5040040

[CIT0172] Turner, B. L., R. D. Rhoades, L. O. Tedeschi, R. D. Hanagriff, K. C. McCuistion, and B. H. Dunn. 2013. Analyzing ranch profitability from varying cow sales and heifer replacement rates for beef cow-calf production using system dynamics. Agric. Syst. 114:6–14. doi:10.1016/j.agsy.2012.07.009

[CIT0173] United States Department of Agriculture (USDA). 2018a. January cattle inventory. Washington, D.C.: USDA. Available fromhttps://downloads.usda.library.cornell.edu/usda-esmis/files/h702q636h/pn89f870n/jw828f69f/catl0122.pdf. [accessed February 27, 2018].

[CIT0174] United States Department of Agriculture (USDA). 2018b. January sheep and goats inventory. Washington, D.C.: USDA. Available from https://downloads.usda.library.cornell.edu/usda-esmis/files/000000018/0p0969332/t722hc50v/SheeGoat-01-31-2018.pdf. [accessed February 27, 2018].

[CIT0175] United States Department of Agriculture (USDA). 2019. 2017 Census of agriculture – United States summary and state data. Volume 1 geographic area series, Part 51, AC-17-A-51. Washington, DC: U.S. Department of Agriculture. Available from https://www.nass.usda.gov/Publications/AgCensus/2017/Full_Report/Volume_1,_Chapter_1_US/usv1.pdf. [accessed June 29, 2020].

[CIT0176] United States Department of Agriculture–Natural Resource Conservation Service (USDA-NRCS). 2003. National range and pasture handbook. Chapter 2. Washington, D.C.: USDA. NRCS. Available from https://www.nrcs.usda.gov/Internet/FSE_DOCUMENTS/stelprdb1043059.pdf. [accessed February 27, 2018.]

[CIT0177] United States Department of Agriculture–United States Forest Service (USDA-USFS). 2018. About rangeland management. Washington (DC): USDA. FS. Available from https://www.fs.fed.us/rangeland-management/aboutus/index.shtml. [accessed February 27, 2018].

[CIT0178] Vaintrub, M. O., H. Levit, M. Chincarini, I. Fusaro, M. Giammarco, and G. Vignola. 2021. Review: Precision livestock farming, automats and new technologies: possible applications in extensive dairy sheep farming. Animal. 15:100143. doi:10.1016/j.animal.2020.100143 33518488

[CIT0179] Vázquez-Diosdado, J. A., V. Paul, K. A. Ellis, D. Coates, R. Loomba, and J. Kaler. 2019. A combined offline and online algorithm for real-time and long-term classification of sheep behaviour: novel approach for precision livestock farming. Sensors. 19:3201. doi:10.3390/s19143201 PMC6679336 31330790

[CIT0180] Veerkamp, R. F. , B. Beerda, and T. van der Lende. 2003. Effects of genetic selection for milk yield on energy balance, levels of hormones, and metabolites in lactating cattle, and possible links to reduced fertility. Livest. Prod. Sci. 83:257–275. doi:10.1016/S0301-6226(03)00108-8

[CIT0181] di Virgilio, A., J. M. Morales, S. A. Lambertucci, E. L. C. Shepard, and R. P. Wilson. 2018. Multi-dimensional Precision Livestock Farming: a potential toolbox for sustainable rangeland management. PeerJ 6:1–23. doi:10.7717/peerj.4867 PMC5984589 29868276

[CIT0182] Wang, J., X. Xiao, R. Bajgain, P. Starks, J. Steiner, R. B. Doughty Q. Chang. 2019. Estimating leaf area index and aboveground biomass of grazing pastures using Sentinel-1, Sentinel-2 and Landsat images. ISPRS J. Photog. Remote Sens. 154:189–201. doi:10.1016/j.isprsjprs.2019.06.007

[CIT0183] Webb, M. J., J. J. Block, A. A. Harty, R. R. Salverson, R. F. Daly, J. R. Jaeger, K. R. Underwood, R. N., Funston, D. P. Pendell, C. A. Rotz, et al. 2020. Cattle and carcass performance, and life cycle assessment of production systems utilizing additive combinations of growth promotant technologies. Transl. Anim. Sci. 4:1–15. doi:10.1093/tas/txaa216 33409468 PMC7770620

[CIT0184] Willet, W. , J. Rockstrom, B. Loken, M. Springmann, T. Lang, S. Vermeulen, T. Garnett, D. Tilman, F. DeClerck, A. Wood, et al. 2019. Food in the Anthropocene: the EAT-lancet commission on healthy diets from sustainable food systems. Lancet. Commissions. 393:447–492. doi:10.1016/S0140-6736(18)31788-4 30660336

[CIT0185] Williams, T., C. Wilson, P. Wynn, and D. Costa. 2021. Opportunities for precision livestock management in the face of climate change: a focus on extensive systems. Anim. Front. 11:63–68. doi:10.1093/af/vfab065 34676141 PMC8527464

[CIT0186] Yu, Y., M. Z. Li, and Y. Fu. 2018. Forest type identification by random forest classification combined with SPOT and multi-temporal SAR data. J. Forest. Res. 29:1407–1414. doi:10.1007/s11676-017-0530-4

[CIT0187] Zengeya, F. M., O. Mutanga, and A. Murwira. 2013. Linking remotely sensed forage quality estimates from worldview-2 multispectral data with cattle distribution in a Savanna landscape. Int. J. Appl. Earth Obs. Geoinf. 21:513–524. doi:10.1016/j.jag.2012.07.008

